# Primate modularity and evolution: first anatomical network analysis of primate head and neck musculoskeletal system

**DOI:** 10.1038/s41598-018-20063-3

**Published:** 2018-02-05

**Authors:** Vance Powell, Borja Esteve-Altava, Julia Molnar, Brian Villmoare, Alesha Pettit, Rui Diogo

**Affiliations:** 1 George Washington University, Department of Anthropology, 2110 G Street NW, Washington, DC 20052 USA; 2 The Royal Veterinary College, Structure and Motion Lab, Hawkshead Lane, Hatfield, Hertfordshire, UK AL97TA USA; 3 Howard University College of Medicine, Department of Anatomy, 520 W Street NW, Washington, DC 20059 USA; 4 University of Nevada, Las Vegas, 4505 S. Maryland Pkwy., Las Vegas, NV 89154 United States

## Abstract

Network theory is increasingly being used to study morphological modularity and integration. Anatomical network analysis (AnNA) is a framework for quantitatively characterizing the topological organization of anatomical structures and providing an operational way to compare structural integration and modularity. Here we apply AnNA for the first time to study the macroevolution of the musculoskeletal system of the head and neck in primates and their closest living relatives, paying special attention to the evolution of structures associated with facial and vocal communication. We show that well-defined left and right facial modules are plesiomorphic for primates, while anthropoids consistently have asymmetrical facial modules that include structures of both sides, a change likely related to the ability to display more complex, asymmetrical facial expressions. However, no clear trends in network organization were found regarding the evolution of structures related to speech. Remarkably, the increase in the number of head and neck muscles – and thus of musculoskeletal structures – in human evolution led to a decrease in network density and complexity in humans.

## Introduction

What transformations occurred in primate evolution to create our modern head and neck morphology and allow us to carry out complex functions such as breathing, speaking, chewing, swallowing and displaying complex facial expressions, while also retaining evolvability? Although there is increasing interest in this question among scientists and the broader public, it remains mainly unanswered; numerous papers and books focus specifically on it, but different biologists and anthropologists have differing views about how to answer it^1^. To address this question, two concepts are paramount: *modularity* and *integration* e.g.^2–7^. Ever since the publication of seminal works on these concepts by Bateson^8^ and Olson & Miller^9^, the idea of an animal’s body as a set of nested parts within parts (modularity) that maintain a level of autonomy to change while still growing and adapting in coordinated ways (integration) continues to gain support as a central mechanism of evolution, e.g.^10,11^. These concepts are tightly linked to questions about *complexity* and *evolvability* (the ability to respond to selective pressure): modularity enables flexibility because the direction and magnitude of evolutionary change among and within parts can vary without sacrificing function, e.g.^12–27^. However, our knowledge of morphological modularity, integration, complexity and evolvability in the primate musculoskeletal system remains limited because of the difficulty of studying the myriad interactions among the body’s hard and soft-tissues. Moreover, most studies have concentrated on hard tissues, in no small part due to the challenge of obtaining soft tissue data and of managing and making sense of complex datasets. For instance, most primate modularity and integration studies focus on cranial bones and teeth (e.g.^2,28–34^). Furthermore, these studies focus mainly on quantitative skeletal traits (e.g. bone length, skull width), although functional and morphological changes in human evolution also involved the reorganization and evolution of traits that are clearly not amenable to these types of measurements (e.g. presence/absence of muscles, bones and articulations^1^). For these reasons, most works on primates refer to concepts such as “module” and “integration” in a rather undefined way without clearly explaining how to quantitatively study them, as stressed by Ross^35^.

Anatomical network analysis (AnNA) of connectivity patterns (e.g. bone-bone and bone-muscle connections) provides an operational, quantitative tool to investigate and generate testable predictions about how morphological organization has changed over primate and human evolution, and therefore about integration, modularity and evolvability, e.g.^36–38^. The use of network theory to study morphological modularity and integration, not only of musculoskeletal structures but also, for instance, of brain tissues, is becoming frequently common in biological studies and has been employed by researchers from many biological fields and working with various animal groups, e.g.^39–43^. AnNA uses topological organization and connectivity relationships (e.g., articulations and attachments) between anatomical structures and/or types of tissues (e.g., bones and muscles) in a way that can be complementary to that provided by morphometric analysis of size and shape. The head and neck of primates and their close relatives are an interesting target for AnNA because they exhibit considerable diversity in the number of muscles and their attachments, which accompanied dramatic functional changes concerning, for instance, the evolution of facial and vocal communication, including the origin of speech^44,45^. We analyzed the anatomical network organization of the musculoskeletal structures of the head and neck in 22 genera representing all major extant primate clades as well as three outgroups: the primate sister-group Dermoptera (colugos), their sister-group Scandentia (tree-shrews), and their sister-group Glires (rodents and lagomorphs), represented respectively by *Cynocephalus*, *Tupaia*, and *Mus* (see Methods below and SI1 and SI2).

We investigated whether there are major differences in the head and neck network organization of these 22 genera, focusing particularly on three major questions concerning primate and human evolution. The first question concerns the evolution of facial communication. Our recent AnNA study of the human head revealed that, in humans, bones and muscles of the middle and lower facial region are grouped into left and right facial musculoskeletal modules, which are mainly functional complexes including structures with different phylogenetic and developmental origins^33,46^. Those results brought new light to the debate on the symmetry/asymmetry of facial expression in humans. Functional, anatomical and medical studies have shown that asymmetrical use of facial muscles in humans is required to display complex facial expressions, e.g.^47,48^. The discovery of such left and right facial (mid/lower face) modules by AnNA thus placed these functional, medical and anatomical observations in a novel quantitative context that may contribute to the understanding of our ability to asymmetrically contract or relax the facial muscles of the mid/lower face and to strike such complex facial expressions. The first question is, therefore: does this division into left and right orofacial muscle modules have a deeper evolutionary origin, or is it unique to humans, thus being potentially related to the finer type/greater number of facial expressions displayed by humans? The second question concerns the increase in the number of facial and laryngeal muscles in human evolution, which led to a greater total number of head muscles in humans than in great apes^44,45^. This trend contrasts with the trend towards a decrease in the number of skull bones in tetrapod evolution (Williston’s Law), which, surprisingly, has been shown to have led to an increase in the density of connections and thus of morphological network complexity in *H*. *sapiens,* e.g.^33,36–38^. In AnNA, density of connections (*D*) is often used as a proxy for the complexity of a morphological structure because the number of functional possibilities and potential functional outcomes increases with the number of connections among parts (for more details, see SI1). The second question is, therefore, whether such a negative correlation between the number of structures vs. the network density and complexity also applies to muscles and thus to musculoskeletal systems as a whole. That is, as the decrease in the number of skull bones was accompanied by an increase in the density of bone-bone connections *(fewer bones -* > *increase of network density and complexity)*, is the increase in number of head muscles also accompanied by a decrease in the density of muscle-bone connections *(more muscles and musculoskeletal structures in total–* > *decrease of network density and complexity)*? The third, related question is: during the ape-human transitions, did the various changes presumably related to the evolution of speech – e.g., increase in number of laryngeal muscles, dropping of the larynx, and rearrangement of pharyngeal structures^1^ – involve major changes in musculoskeletal connectivity, modularity and integration? The present study can therefore contribute substantially to a comprehensive understanding of the evolution of morphological modularity, integration and complexity in the primate/human head and neck, and to biological and physical anthropology and evolutionary biology in general.

## Results and Discussion

Before describing and discussing our results we provide here a very brief summary about which head muscle groups are present in humans - as the same muscle groups are also found in all the other taxa included in this paper - in order to allow those readers that might not have a deep knowledge about myology to follow the text and understand the figures more easily. The *mandibular muscles* are generally innervated by the Vth (trigeminal) nerve, and include the mastication muscles masseter, temporalis, lateral pterygoid, medial pterygoid, mylohyoid, and anterior belly of the digastric, the ear muscle tensor tympani and the tensor veli palatini that lies in the pharyngeal region. The *hyoid muscles* are innervated by the VIIth (facial) nerve and include the posterior belly of the digastric and the stylohyoid, which are suprahyoid structures, the ear muscle stapedius, and all the facial expression muscles (platysma, risorius, occipitalis, posterior auricular, external auricular muscles, zygomaticus major, zygomaticus minor, frontalis, anterior auricular, superior auricular, temporoparietalis, orbicularis oculi, depressor supercilii, corrugator supercilii, levator labii superioris alaeque nasi, procerus, buccinatorius, levator labii superioris, nasalis, depressor septi nasi, levator anguli oris, orbicularis oris, depressor labii inferioris, depressor anguli oris, mentalis). The *branchial muscles* include the stylopharyngeus innervated by the IXth (glossopharyngeal) nerve - which forms part of the long constrictors of the pharynx - plus the neck muscles trapezius and sternocleidomastoideus innervated by the XIth (accessorius) nerve, and the laryngeal (cricoarytenoideus posterior, thyroarytenoideus, vocalis, cricoarytenoideus lateralis, arytenoideus transversus and obliquus) and pharyngeal (superior, middle and inferior pharyngeal constrictors and cricothyroid, levator veli palatini, salpingopharyngeus, patalopharyngeus and musculus uvulae) muscles innervated by the Xth (vagus) nerve. The extraocular muscles (levator palpebrae superioris plus superior, inferior, medial and lateral recti and superior and inferior obliquii) are innervated by nerves III (oculomotor), IV (trochlear) and VI (abducens) of the head. Lastly, the hypobranchial muscles (geniohyoid, genioglossus, part of intrinsic tongue muscles, hyoglossus, styloglossus, omohyoid, sternohyoid, sternothyroid and thyrohyoid), which are derived from the anterior somites of the body and not from head mesoderm and are therefore intrusive elements of the head, are innervated by the XIIth (hypoglossal) nerve plus cervical spinal nerves. For a more detailed overview on all these muscle groups and their subgroups, as well as their functions, comparative anatomy, homologies and evolution, see Diogo & Wood book on the primate musculature^49^.

### Network parameters of head and neck bones-only networks

AnNA allows the quantitative characterization of broad patterns of structural integration and modularity regardless of changes in number, size, and shape of parts. We specifically compared the values of a set of six network parameters within the taxa analyzed: number of nodes (N), number of links or connections (K), density of connections (D), average clustering coefficient (C), average shortest path length (L), and heterogeneity (H). Whereas N and K are useful for comparing the number of constitutive anatomical parts, parameters D, C, and L help to identify patterns of complexity and integration (reviewed in Rasskin-Gutman and Esteve-Altava^38^; see SI1 for more details). In short, higher values of D and C and lower values of L are characteristic of greater complexity and integration; higher values of H are characteristic of greater anisomerism. Our AnNA results show that the number of nodes (bones) ranges from 37 in *Homo* to 41 in *Lemur* and lorises. This variation is primarily dependent on the presence or absence and fusion or separation of specific facial bones. For instance, *Homo* alone lacks a separated premaxilla, but like some other taxa it has fused mandibles. The number of skeletal connections in humans is among the lowest within primates (K = 72), with only *Pan* (K = 70) and *Pongo* (K = 69) exhibiting lower K, but the network density (D) and thus complexity (see above) of *Homo* is greater than that seen in any other taxon we studied. Most of the connections that contribute to the networks’ densities involve the temporal bone (squamosal in *Mus*), which articulates with many surrounding cranial bones. The clustering coefficient (C; i.e., the average of the sum of connections between all neighbors of each node with respect to the maximum number possible) also shows values that are nearly unique to each taxon and that do not seem to follow a phylogenetic trend, except that the Old World Monkeys (OWM, or cercopithecids) *Macaca*, *Papio*, and *Colobus* exhibit identical values of C. Worth noting is that *Homo* and *Mus* also exhibit identical clustering coefficient values. The average shortest path length (L; i.e., the average of the minimum distance between all nodes in the network in terms of connections, with each connection representing 1 length unit) is similar among all taxa (roughly between 2.7 and 2.8) except for *Gorilla* and *Tarsius* (2.65), and *Mus* (2.3). This consistency of values is likely the result of the conserved number of cranial bones across these genera. The consistency of cranial elements also impacts the observed values of heterogeneity, the variability of which displays some apparent phylogenetic signal among our taxa. Further discussion regarding the links between number of skeletal structures and network density and complexity within primate evolution will be presented below (see also Tables SI1–3; Figs SI1–2).

### Network parameters of head and neck muscle-bone networks

Among all taxa, the number of nodes (bones + muscles) ranges from 141 in *Saimiri* to 161 in *Tupaia* and 175 in *Mus*. Importantly, *Homo* has the highest value for N (157) among all primates; i.e., despite the lower number of skeletal structures in humans (see Section above), the fact that humans have many more head and neck muscles than any other primate leads to a higher number of head and neck musculoskeletal structures overall. Interestingly, regarding network density (D) and thus complexity, the three taxa with the most musculoskeletal structures – *Tupaia*, *Mus* and *Homo* – are those with the lowest D (0.029). With respect to the clustering coefficient (C), there is a phylogenetic pattern in which anthropoids exhibit the highest values in general. An inverse, though much weaker, pattern is observed regarding the shortest path length (L), in which there is a tendency for anthropoids and *Tarsius* to exhibit lower values than do, e.g., lemuriforms and *Mus*. Finally, heterogeneity (H) of head and neck musculoskeletal networks displays less consistency than the other network parameters. Many of the musculoskeletal network parameters are similar among the most closely related taxa, as would be expected if the presence and arrangement of bones and muscles were phylogenetically constrained. New world monkeys (NWM) have the absolute fewest nodes, smallest numbers of connections, and shortest path lengths, but intermediate to high numbers of connections and values for density, clustering coefficient, and heterogeneity. Hominoids (apes + humans) display intermediate to low numbers of nodes, intermediate numbers of connections and density of connections, and among the shortest path lengths. Excluding the intermediate *Hylobates*, hominoids have some of the greatest clustering coefficient values, with *Pan* exhibiting the absolute greatest values. OWM have intermediate values for all parameters, except for the clustering coefficient in which they display some of the highest values, consistent with anthropoids in general. The non-primates *Cynocephalus* and *Tupaia* are intermediate in many cases, but regarding the density of connections *Cynocephalus* is toward the higher end of the spectrum, whereas *Tupaia* and *Mus* have the absolute lowest values together with *Homo*, as noted above, as will be discussed in the Sections below (see also Table SI1–1; Fig. SI1–1).

### Anatomical modules of head and neck muscle-bones networks

**Figure 1:**
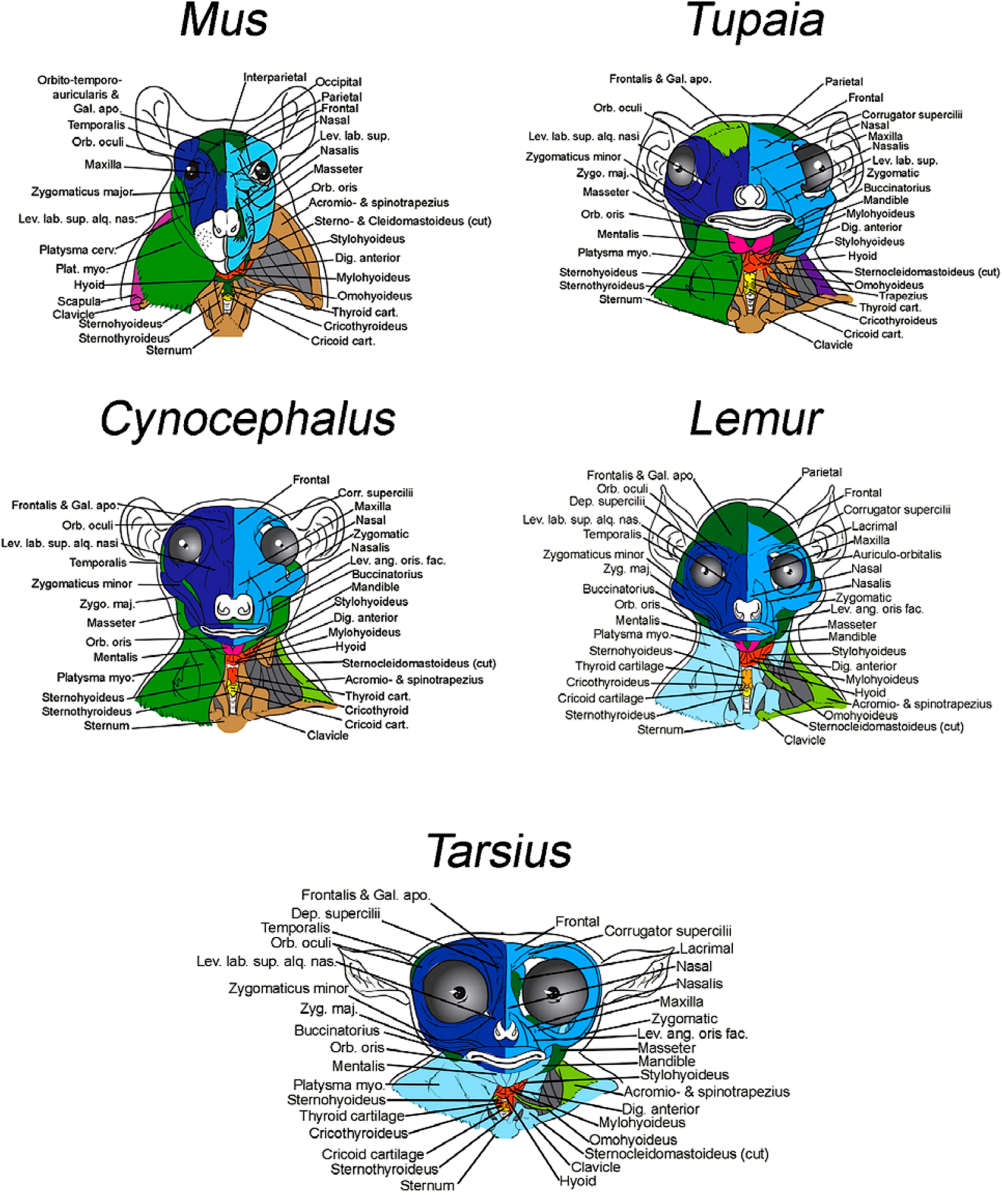
Musculoskeletal modules of non-anthropoid heads and necks identified using AnNA. For each taxon, as well as for the other taxa that were analyzed for the present work from the clade to which the taxon belongs, modules, color codes, and constituent anatomy are provided in SI1 Tables 4–7 and 11. *Mus*, 20 modules; *Tupaia*, 19 modules; *Cynocephalus*, 15 modules; *Lemur*, 15 modules; *Tarsius*, 16 modules. cart., cartilage; Corr. supercilii, Corrugator supercilii; Dep. supercilii, Depressor supercilii; Dig. anterior, Digastricus anterior; Gal. apo, Galea aponeurotica; Lev. ang. oris fac., Levator anguli oris facialis; Lev. lab. sup., Levator labii superioris; Lev. lab. sup. alq. nas., Levator labii superioris alaeque nasi; Orb. oculi, Orbicularis oculi; Orb. oris, Orbicularis oris; Plat. myo., Platysma myoides; Zygo. maj., Zygomaticus major.

**Figure 2:**
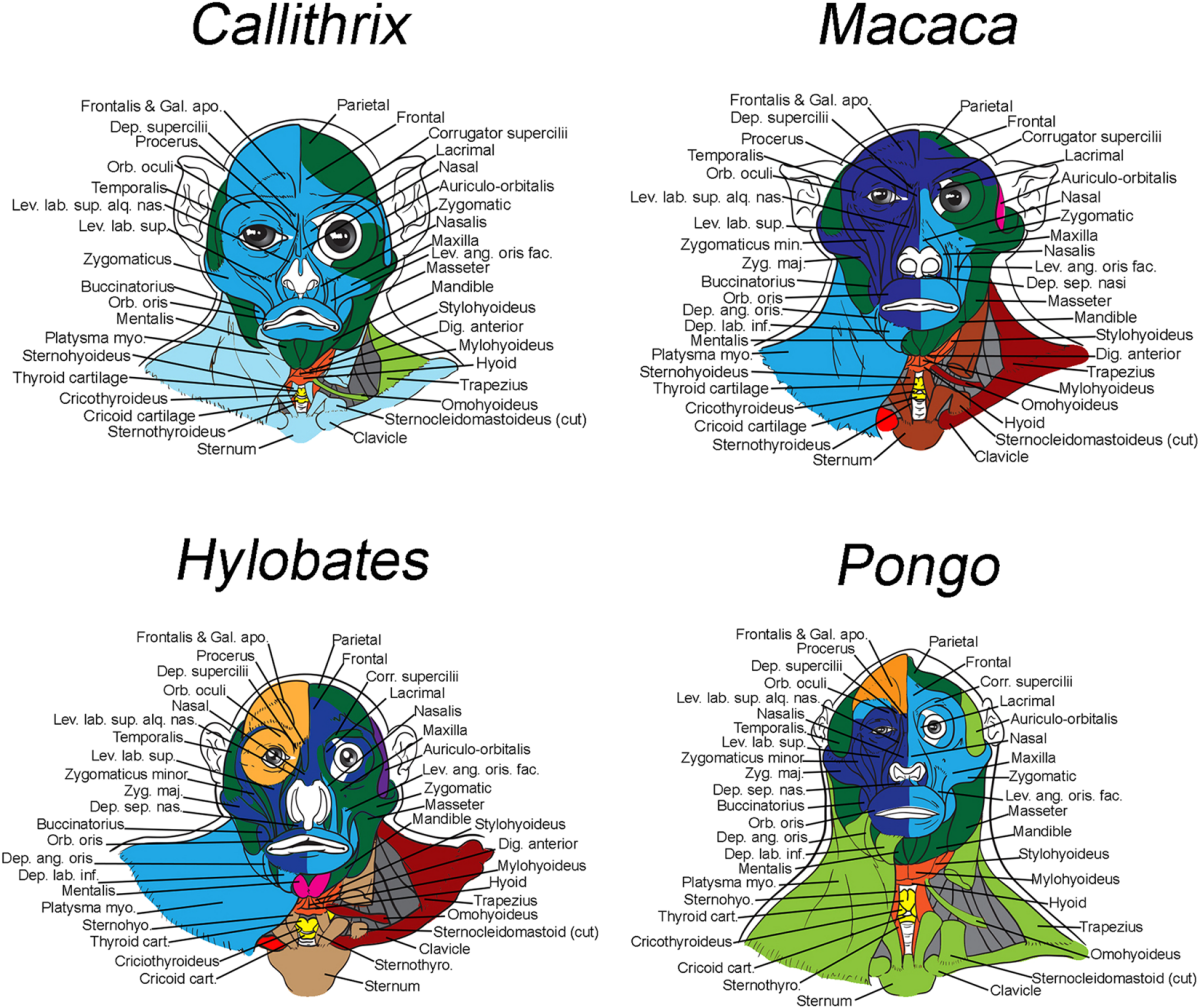
Musculoskeletal modules of monkey and non-African ape heads and necks identified using AnNA. For each taxon, as well as for the other taxa that were analyzed for the present work from the clade to which the taxon belongs, modules, color codes, and constituent anatomy are provided in SI1 Tables 12 and 19–21. *Callithrix*, 12 modules; *Macaca*, 18 modules; *Hylobates*, 17 modules; *Pongo*, 12 modules. Dep. ang. oris., Depressor anguli oris; Dep. lab. inf., Depressor labii inferioris; Dep. sep. nasi, Depressor septi nasi; Sternohyo., Sternohyoideus; Sternothyro., Sternothyroideus; Zygomaticus min., Zygomaticus minor (for other abbreviations, see Fig. 1).

**Figure 3:**
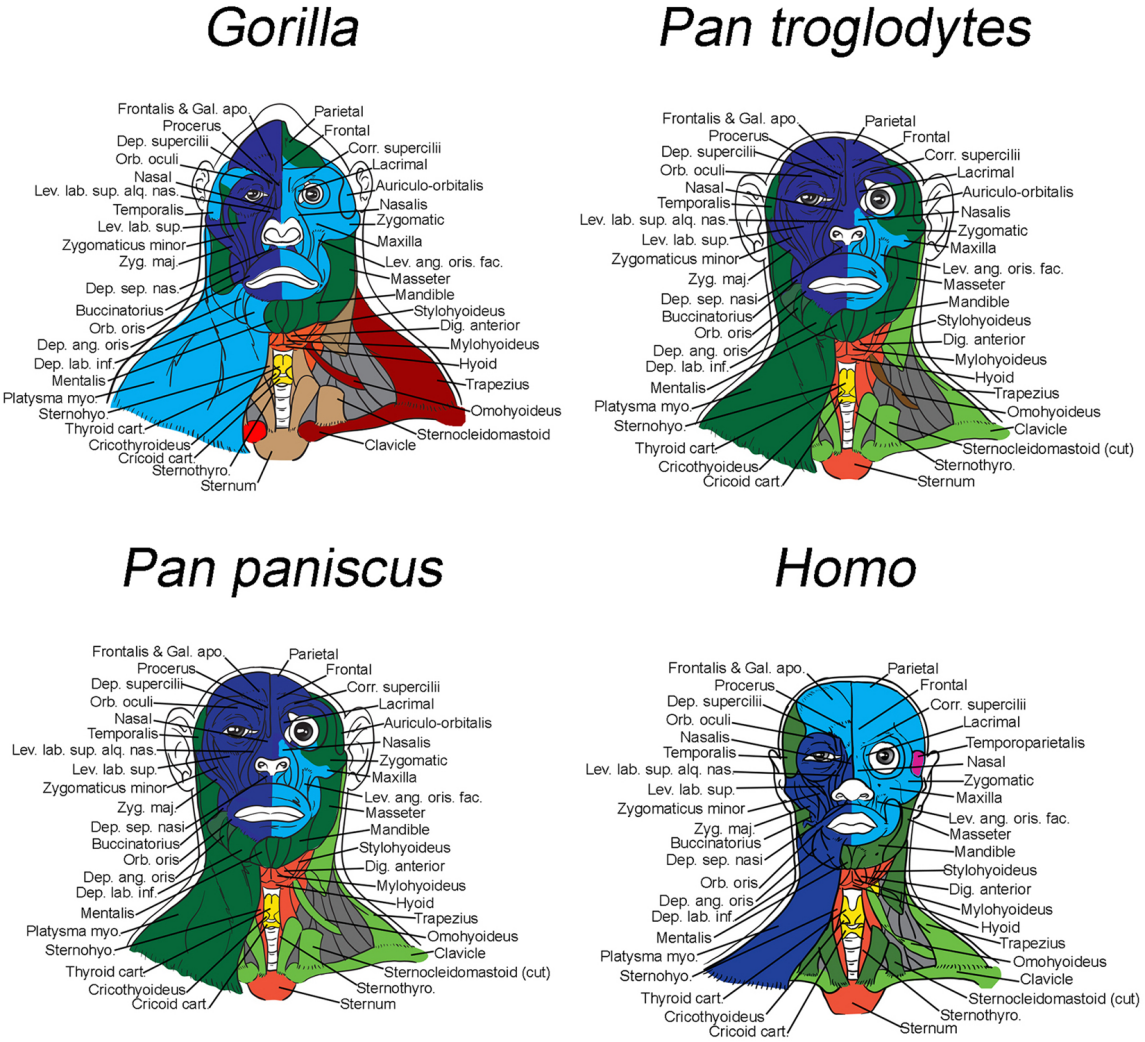
Musculoskeletal modules of African ape and human heads and necks identified using AnNA. For each taxon, modules, color codes, and constituent anatomy are provided in SI1, Tables 22–25. *Gorilla*, 17 modules; *P*. *troglodytes*, 12 modules; *P*. *paniscus*, 11 modules; *Homo*, 11 modules. (for other abbreviations, see Figs 1 and 2).

We recently published a paper focusing exclusively on the anatomical modules of the head bones-only networks of the taxa included in the present study^33^. Therefore, in this Section we will focus instead on the modules of the muscle-bone (i.e. musculoskeletal) networks, which are, moreover, those that can be discussed in a more integrative, comprehensive functional and/or macroevolutionary context. Readers interested in knowing more details about all the skeletal modules of all taxa analyzed should refer to Tables SI1–26 to SI1–48. Excluding a few genera (detailed below), all taxa maintain some version of the following major musculoskeletal modules. One is the ‘neurocranial’ module (shown in dark green in Figs 1–3**)**, which typically includes neurocranial bones and facial, masticatory and/or pharyngeal muscles. Two other modules are the right and left facial modules (shown in dark and light blue respectively), typically including facial bones and facial expression muscles. Another one is the ‘suprahyoid and tongue’ (shown in orange) module that may include structures of other anatomical regions. A ‘true vocal fold movement’ module is often combined with a ‘laryngeal movement’ module (shown in yellow): as their names indicate, these modules include structures directly related to the movement of the true vocal folds (e.g. arytenoid cartilages and muscles moving them) and of the larynx (e.g. cricoid and/or thyroid cartilages and muscles moving them). Lastly, there are often ‘postcranial’ modules (often shown in light brown), which in a few taxa are separated into right and left postcranial modules (often shown in red and dark red, respectively). Although considerable variation exists in the constituent anatomy of each specific module (see Figs 1–3 and Tables SI1, 2 to SI1–24, as well as text below, for more details) the fact that some version of these modules is found in most taxa, including the non-primate ones, indicates that AnNA is in fact a robust method to study the modularity of musculoskeletal structures (Figs 1–3; Tables SI1, 2 to SI1–24.

Regarding the different clades analyzed, *Mus* (representing Glires, the sister-group of the other taxa analyzed) has a ‘laryngeal and true vocal fold movement’ module, a large ‘neurocranium & facial, hyoid, pharyngeal and tongue muscles’ module that is a mix of bones and muscles related to very different functions, as its name indicates, a ‘postcranial & infrahyoid muscles’ module that includes the sternum, clavicles, and various neck muscles, a ‘dorsal postcranial module’ including the vertebrae and the trapezius muscles, a ‘suprahyoid muscles’ module, and the pharyngeal and facial muscles distributed among numerous other modules, including the ‘right and left facial’ modules (see Section above; Fig. 1; Tables SI1–3). Of all major modules of *Mus*, the only ones that are very similar in the tree-shrew *Tupaia* with respect to the specific structures that they include are the ‘laryngeal and true vocal fold movement’, the ‘postcranial & infrahyoid muscles’ and the ‘left facial’ and ‘right facial’ modules (Fig. 1; Tables SI1–4). In contrast, *Tupaia* is very similar to the dermopteran *Cynocephalus*, sharing with the latter taxon a very similar composition of not only these four modules but also the ‘suprahyoid, pharyngeal and tongue’ module, the ‘neurocranium & facial, masticatory and pharyngeal muscles’ module, and ‘mentalis’ module (Fig. 1; Tables SI1–5). This is, therefore, a case in which AnNA clearly seems to be recovering a phylogenetic pattern, because in fact many of these *Tupaia* and *Cynocephalus* modules are also very similar in their composition to those of strepsirrhines such as *Lemur* (Fig. 1; Tables SI1–6). Moreover, *Lemur*, as well as all other strepsirrhines included in the analysis (Tables SI1–6 to SI1–9) also has some modules not seen in non-primate taxa, such as a ‘true vocal fold movement’ module that exclusively includes structures related to the movement of the vocal folds and that is shared with many other primate taxa; i.e., in these taxa these structures are *not* part of a larger ‘laryngeal and true vocal fold movement’ module (see Section above), as is the case in the non-primate taxa.

The presence of a separate module exclusively related to the movement of the vocal cords, which only occurs in primates, could well be related to their enhanced display of vocal communication. However, this module is not universally present as a separate module in primates, as we discuss below. This module is present in *Tarsius* (Fig. 1; Tables SI1–10), which in turn also has other modules that are not found in *Lemur* but are present in other primates, such as a module including both the supra- and infrahyoid, as well as tongue, muscles (‘supra-infrahyoid and tongue’ module), further supporting the idea that AnNA is recovering phylogenetic patterns, which may well represent synapomorphies (e.g., ‘true vocal fold movement’ module only present in primates, module including both supra and infrahyoid muscles only present in *Tarsius* + anthropoids, and so on). In fact, apart from this major difference between *Tarsius* and strepsirrhines such as *Lemur*, most other modules of these taxa are very similar in composition. The few exceptions concern small differences, such as the fact that in *Tarsius* the ‘postcranial module’ does not include the clavicles, and that the module including the sternum includes facial muscles as in *Lemur* but, contrary to *Lemur*, also includes the clavicles and mandible and does not include infrahyoid muscles.

Similarly, within anthropoids, the major modules of NWM (the sister-group of other anthropoids) such as *Callithrix* (Fig. 2; Tables SI1–11) are broadly similar to those found in *Tarsius*, with two major differences directly related to the smaller number of modules found in NWM: 1) the ‘laryngeal movement’ module and ‘true vocal fold movement’ module are integrated into a single ‘laryngeal and true vocal fold module movement’ module, and the ‘left facial’ and ‘right facial’ modules are integrated into a single ‘main facial’ module. Interestingly, there is a lot of variation within the NWM: e.g., most modules of *Saimiri* (Tables SI1–12) are much more similar to those of *Lemur* than to those of *Callithrix*, but both *Saimiri* and *Callithrix* share the ‘main (left + right) facial’ module. As this ‘main facial’ module is also present in *Pithecia* (Tables SI1–14), it is likely that its presence is in fact plesiomorphic for NWM (at least of those included in our AnNA) and was secondary lost in *Aotus*. In fact, a similar ‘main facial’ module is also present in the OWM genera *Colobus* (Tables SI1–15), which is the sister-group of the other OWM included in the AnNA, and *Cercopithecus* (Tables SI1–16), which is the sister-group of *Macaca* + *Papio*. Therefore, the presence of a ‘main facial’ module could well be a synapomorphy of anthropoids that was secondary lost/changed in *Aotus*, in *Macaca* + *Papio* and in hominoids. Studies including more taxa are needed to test this hypothesis. In addition, more detailed studies on the facial expression displays of both OWM and NWM, ideally including FACS (Facial Action Coding System: see, e.g., Parr *et al*.^50^) and analysis of both captive and wild animals, are needed to investigate whether having separate left and right facial modules vs. a main facial module might be related to an increased or decreased ability to display complex facial expressions; see also Section below for further discussion on these facial modules.

Regarding hominoids, *Hylobates* (Fig. 2; Tables SI1–19) has, interestingly, an increased number of modules composed almost exclusively of muscles of facial expression and excluding bony structures. While such a configuration could indicate modularity of facial expression and thus greater finesse in facial displays, it may instead indicate reduced cooperation of facial movement and less coordinated facial expressions, as will also be further discussed below. Regarding not only the number but also the specific composition of each module, humans are much more similar to our closest living relatives (common chimps and bonobos) than to gorillas and orangs (Figs 2, 3; Tables SI1–20 to SI1–24), further supporting the idea that AnNA can be an effective tool to detect phylogenetic signals. The musculoskeletal modules of common chimps and bonobos are particularly similar to each other, as expected, but in common chimps there is a unique, peculiar module that includes the left scapula and omohyoideus (shown in brown in Fig. 3), and the left buccinatorius is not part of the left facial module (shown in dark green in Fig. 3). Interestingly, concerning these two features bonobos are similar to modern humans, indicating that bonobos are probably a better model for the human-chimp ancestor than common chimps are, regarding not only the presence/absence and overall configuration of their muscles^51,52^ but also the details of their head and neck modules.

### Evolution of head and neck musculoskeletal modules and modularity

So, what does our AnNA tell us about the three main questions raised in the first Section of this paper, and about the general patterns concerning the macroevolution of head and neck musculoskeletal modules and modularity in the taxa analyzed? Regarding the question of whether the presence of left and right facial modules is a unique human feature or a more ancestral trait, our AnNA results show that the presence of these two modules is clearly plesiomorphic for primates, as explained above and as shown in Figs 1–3. Most taxa analyzed by us, primate and otherwise, exhibit such left and right facial modules, the exceptions being the OWM *Colobus* and *Cercopithecus* and the NWM *Pithecia*, *Saimiri* and *Callithrix*, which display instead a ‘main facial’ module, as noted above. Also as explained above, the presence of such a ‘main facial’ module is clearly derived within primates and may be a synapomorphy of anthropoids. Importantly, a feature unique to anthropoids is that the right and left facial modules do not include only right and left structures, respectively. That is, in all anthropoids there is either a ‘main facial’ module with both left and right structures or separate left and right modules that are highly asymmetrical; i.e., the left module includes at least some right facial bones and/or muscles and/or the right module includes at least some left ones (Figs 2 and 3; Tables SI1–11 to SI1–24). In fact, the only exception among the 14 anthropoid taxa included in our AnNA is *Aotus*, which, like non-anthropoids, has left and right modules including only left and right structures. Therefore, the enhanced complexity of facial expressions, including asymmetrical use of structures of the two sides of the face for displaying highly complex expressions as reported in humans, is very likely related to such modular integration/asymmetry of the facial modules rather than to a better defined modular left vs right symmetry such as that seen in non-anthropoids (compare Fig. 1 with Figs 2, 3). It is important to note that we are not referring to an asymmetry of the left vs. right musculature per se, but instead to an asymmetry of the left and right facial *network* *modules* that include many of these muscles. That is, it is not the muscles themselves, but instead the *network modules* made by the contacts/fusions between them and/or by their attachments to bones that are asymmetrical.

The only facial feature we identified that might distinguish humans from other taxa is that in humans 3/4 of all muscles of facial expression (75%, i.e. 36/48) are part of these left and right asymmetrical modules (Fig. 3; Tables SI1–24). In contrast, in chimps almost a third of the facial muscles (30%; i.e., 12/44 in common chimps) are part of an unspecialized, bilateral module that also includes inner ear, masticatory and pharyngeal muscles (shown in dark green in Fig. 3); only 66% (29/44) and 68% (30/44) of the facial muscles are included in the left and right facial modules in common chimps and bonobos, respectively (Fig. 3; Tables SI1–22, SI1–23). This is particularly interesting because concerning both the anatomy and the networks of the brain, humans are also more asymmetrical than chimps and other primates^53^. In *Pongo*, the percentage of muscles included in these left and right asymmetrical facial modules is even smaller: only 59% (24/46). Therefore, it seems that there is a trend towards more integration within these two asymmetrical facial modules during primate and human evolutionary history, as predicted by Lieberman^1^ for the head as a whole. That is, more facial structures are included in fewer modules. An emblematic example of this trend is *Hylobates*: the facial muscles of these lesser apes, which by no means display more facial expressions or more complex ones than great apes and or humans do^54–59^, are dispersed among eight modules (Fig. 2; Tables SI1–19), and the left and right facial modules include only 60% (28/46) of the facial muscles, a percentage similar to that found in orangutans. The exception among apes is *Gorilla*, in which 81% (36/44) of the facial muscles are included in the right and left facial modules, a percentage even higher than that of humans, which have the second highest percentage among all primate and non-primate taxa analyzed (Fig. 3; Tables SI1–21). One can clearly see a general trend towards an increase in percentage in the lineage leading to great apes; e.g., in the OWM *Macaca* the percentage is 72% (33/46) and in the NWM *Callithrix* it is 63% (24/38) (Fig. 2), while in *Tarsius* it is 52% (22/42), in *Lemur* 54% (24/44), in *Cynocephalus* 66% (28/42), in *Tupaia* 54% (25/46) and in *Mus* only 31% (Fig. 1; Tables SI1–3 to SI1–18). *Mus* is an illustrative example because its facial muscles are distributed among seven modules having 16, 2, 8, 7, 7, 2 and 2 facial muscles apiece. That is, in *Mus* the left and right facial modules contain only 14 of the 44 (i.e. 31%) facial muscles. Moreover, the facial muscles in the left and right facial modules are not exclusively grouped with each other, as these modules also include non-facial muscles such as the temporalis and masseter. Therefore, the facial modules of *Mus* are both very small and unspecialized (Fig. 1; Tables SI1–3).

Regarding the question of whether there was a clear change in the whole network organization, connectivity and/or modularity in human evolutionary history related to the evolution of speech, the answer seems to be negative. At first sight, when one compares humans with chimps, there is a clear difference in the sense that humans have two separate, specialized bilateral modules exclusively related to the movements of the larynx (i.e. of cricoid-thyroid cartilages, shown in yellow in Fig. 1) and of the true vocal folds, respectively (i.e. including only the arytenoid cartilages and the muscles attaching to them: see Tables SI1–22 to SI1–24). However, this example illustrates the importance of including more taxa and having a broader phylogenetic context. Many other taxa analyzed also have separate ‘laryngeal movement’ and ‘true vocal fold movement’ modules (see above and Tables SI1–3 to SI1–24), so it is difficult to argue that the presence of these two modules in humans was crucial for the acquisition of speech. Instead, this example seems to show how even major changes in function, including those that involved substantial changes in anatomy – e.g., production of speech and the associated descent of the larynx and changes in the pharynx^1^ – can occur without profound changes to the network organization and modularity of the whole system. That is, a certain network organization might be plastic enough to accommodate such functional and anatomical modifications without being substantially modified.

Lastly, we raised the following question above: as the decrease in number of skull bones was accompanied by an increase in density of bone-bone connections in tetrapod and human evolution *(fewer bones -* > *increase of network density and complexity)*, is the increase in number of head muscles also accompanied by a decrease in the density of muscle-bone connections in human evolution *(more muscles and musculoskeletal structures in total–* > *decrease of network density and complexity)*? The answer is clearly positive. As explained above, humans have the fewest skeletal elements (N = 37) of all 22 taxa analyzed, and accordingly they have higher skeletal network density (D) and thus network complexity (D = 0.108) (Tables SI1–25). Despite having fewer skeletal elements, the total number of muscles + bones (N = 157) is higher than any other primate taxon because humans have many more head and neck muscles in total than any other primate (Table SI1–1). Accordingly, humans have the lowest musculoskeletal network density and complexity among primates (D = 0.029) (Table SI1–1). In fact, this negative link between the number of structures and the network density and complexity seems to apply not only to the skeletal and musculoskeletal structures of humans, but also to the other taxa analyzed. For instance, the two (non-primate) taxa that rank among the top three with humans in number of musculoskeletal structures, *Mus* (N = 175) and *Tupaia* (N = 161), also rank among the bottom three with humans in network density (D = 0.029 as in humans) (Table SI1–1). On the other extreme, the taxon with the fewest musculoskeletal structures (*Saimiri*, N = 141) is the one with the highest network density and complexity (D = 0.035) (Table SI1–1).

Therefore, a take-home message is that an operational, quantitative study of the evolution of primate and human modularity does not support the idea that humans are more complex than other primates, at least with respect to network organization and network density. In fact, the present study provides an example of explicitly quantitative works that contradict *a priori* expectations; e.g., that the evolution of speech would be related to a major re-organization of the topological and connectivity patterns of the head and neck structures (see above). The use of new quantitative methods, including network theory, should thus complement that of more traditional methods to study modularity, integration and complexity, such as morphometrics, to provide a more comprehensive understanding of what makes us humans, and of the evolution of uniquely human traits related to facial and vocal communication such as the ability to speak and to display exceptionally complex facial expressions. Therefore, we hope that this work will pave the way for future studies on the musculoskeletal network organization within specific primate subgroups and comparisons with other mammals and tetrapods, as well as how AnNA can be complemented with such more traditional methods of investigating integration and modularity to reach a more integrative knowledge about the evolution of heads and necks.

## Methods

The detailed description of the specific methods used for the AnNA is too long to be provided in the main text, so it is given in the first Section of SI1. The skeletal data used to code the matrices of the 22 taxa included in the present study were compiled from a dataset provided in a previous paper^33^ that was modified and expanded by BV and AP to exclude the nasal conchae and to include structures from the hyoid and laryngeal apparatuses (see SI2). The muscle data and muscle-bone connectivity data coded in those matrices were provided in Diogo & Wood’s^44,49^ publications, which were based on original dissections and an extensive review of the literature. Multiple specimens of each taxon are therefore represented in this dataset, and the most common configuration reported for each taxon was coded in the network matrices. As explained in detail in Diogo & Wood^44,49^, concerning sample size and the issue of anatomical variability, two points should be stressed. The first point is that the number of non-human primates, including apes, dissected by Diogo & Wood^44,49^ is actually very large even by the standards of comparative anatomical studies of animals that are more easily available for dissections. The second point is that the sample size included in their datasets refers to the specimens dissected by them plus the total number of specimens reported in the numerous publications by other authors that they reviewed. That is, when we code the presence of a certain muscle and/or its attachment to a certain bone in a taxon X, this is because these traits are present in ≥than 50% of the dissected specimens of that taxon, considering all the information available. For instance, regarding the presence/absence of certain muscles, we consider information obtained from dissections of more than 20 hylobatid specimens, 19 orangutans, 25 gorillas, 38 common chimps and 11 bonobos in total, just within apes (for more details, see Diogo & Wood^44,49^). So, in this specific example, for a certain muscle the total sample size, just for apes, is 123 specimens. As for most of these specimens there is information about both sides of the body, we have information, just for apes, for >200 cases, about whether a certain muscle is present or not on one side of the body. Such a total sample size is therefore very high when compared to other anatomical studies, and particularly to studies based on soft tissue characters, and these numbers therefore show that the coding of the matrices for the 22 taxa very likely does reflect the most common condition found in each of those taxa.

### Data availability

All data generated or analyzed during this study are included in this published article (and its supplementary information files).

## Electronic supplementary material

supplementary information

matrices
