# Supplementary material for: Primate modularity and evolution: first anatomical network analysis of primate head and neck musculoskeletal system
Source: Sci Rep. 2018 Feb 5;8:2341. doi: 10.1038/s41598-018-20063-3 (PMC5799162; doi:10.1038/s41598-018-20063-3)
Supplement: Supplementary file 1 — supplementary information [file 41598_2018_20063_MOESM1_ESM.doc]

# Primate modularity and evolution: first anatomical network analysis of primate head and neck musculoskeletal system Vance Powell^1^, Borja Esteve-Altava^2,3^, Julia Molnar^3^, Brian Villmoare^4^, Alesha Pettit^4^, Rui Diogo^3^

1. George Washington University, Department of Anthropology, 2110 G Street NW, Washington, DC 20052
2. The Royal Veterinary College, Structure and Motion Lab, Hawkshead Lane, Hatfield, Hertfordshire, UK AL97TA
3. Howard University College of Medicine, Department of Anatomy, 520 W Street NW, Washington, DC 20059
4. University of Nevada, Las Vegas, 4505 S. Maryland Pkwy., Las Vegas, NV 89154

SI1 - Primate head networks

# 1- Details about methods

# Network modeling

We built unweighted, undirected network models of the musculoskeletal anatomy of the heads of 18 primate species and the three out-group species (see main text). These anatomical networks formalize the anatomical organization of the head as nodes connected by links. The codification of musculoskeletal networks of heads followed that of a previous work on the human head^1^. Here, links represent physical joints among skeletal and muscular elements (e.g., articulations, attachments, and blending). Every contact present between two nodes is coded as 1 and the absence of contact between two bones is coded as 0 in the adjacency matrix that codes the network model. We also analyzed the skeletal and muscular components separately and created skeletal and muscular networks to this end. Skeletal networks include bones and cartilages (as nodes) connected by their articulations (as links). Anatomical networks were coded as adjacency matrices in Excel sheets and analyzed in *R*^2^ using the package *igraph*^3^.

## Network Parameters

We compared the overall anatomical organization of each body part using 6 network parameters: number of nodes (*N*), number of connections (*K*), density of connections (*D*), average clustering coefficient (*C*), average shortest path length (*L*), and heterogeneity of connections. *N* and *K* account for the number of anatomical structures modeled. *D* is the number of actual connections with respect to the maximum possible. *D* is often used as a proxy for the complexity of a morphological structure, because the more connections among parts, the more functional possibilities, and more potential functional outcomes. *C* is the average of the sum of connections between all neighbors of each node with respect to the maximum possible, and it measures the number of triangular loops or motifs in the network. *C* is used as a proxy for the relative amount of biological inter-dependence between the parts. *L* is the average of the minimum distance between all pairs of nodes in the network; distance is measured in number of connections, each having one unit length. *L* is used as a proxy for effective proximity (e.g., ability to work together) among anatomical parts. Finally, *H* is the ratio between the standard deviation of connections per node and the mean number of connection per node, which provides an estimate of the irregularity of the network. *H* is used as a proxy of anisomerism, and how different or non-homogeneous are the parts that compose the morphological structure in their number of connections. Further details on the mathematical description and morphological interpretation of these parameters have been given elsewhere ^4–8^. We used functions from the package igraph to quantify these parameters.

## Phylogenetic Mapping and Ancestral State Reconstruction

We mapped the network parameters on a consensus calibrated phylogeny of primates and their closest living relatives ^see 8^, adding *Mus musculus* as an additional out-group^9^. We reconstructed the ancestral state of each network parameter by estimating states at internal nodes using maximum likelihood, interpolating the states along each branch as in Felsenstein^10^. Reconstruction and mapping was performed using the function *contMap* of the package phytools^11^ in R.

## Community Detection

We delimited the modules of the anatomical networks using a standard random walk algorithm, using the function *cluster_walktrap* of igraph. The heuristics of this algorithm is the idea is that short random walks (we used random walks of 3 steps) tend to concatenate nodes within the same module^12^. This allows us to find groups of nodes (modules) more densely connected among themselves than to nodes outside of the module. The quality of the identified partitions is evaluated using the optimization function modularity (*Q*) defined by Newman and Girvan^13^, which is commonly used to assess whether the partition identified by a community detection algorithm is better that what is expected at random. *Q* will be close to 0 if the number of links within modules is no better than that expected at random; *Q* will be closer to 1 if the modules identified deviate from what is expected for a random network. According to Newman and Girvan’s^13^ observations, usual values of strongly modular networks range between 0.3 and 0.7. The expected error of *Q* was calculated using a jackknife procedure, where every link is an independent observation.

Additionally, we performed a two-sample Wilcoxon rank-sum test on the internal *vs.* external connections of every module to estimate their statistical significance. According to the general definition of a module as a group of nodes highly connected among themselves and poorly connected to nodes in other groups, we expect internal connections to be significantly higher than external connections (H_0_: *K*_internal_ = *K*_external_; H_a_: *K*_internal_ *> K*_external_). Lower p-values tell us to reject H_0_, and hence we can assume the alternative hypothesis that the nodes of the module are more connected among themselves than to other nodes outside the module. In other words, the module identified is not expected by a random grouping of nodes. Note, however, that the accuracy of this test will depend on the sample size; that is, the number of nodes in the module. Smaller modules will render unreliable p-values.

References

1. Esteve-Altava, B., Diogo, R., Smith, C., Boughner, J. C. & Rasskin-Gutman, D. Anatomical networks reveal the musculoskeletal modularity of the human head. *Sci. Rep.* **5,** 8298 (2015).

2. R Core Team. *R: A language and environment for statistical computing.* (R Foundation for Statistical Computing, 2016).

3. Csardi, G. & Nepusz, T. The igraph software package for complex network research. *InterJournal Complex Syst.* **1695,** 1–9 (2006).

4. Esteve-Altava, B., Marugán-Lobón, J., Botella, H. & Rasskin-Gutman, D. Network models in anatomical systems. *J. Anthropol. Sci.* 175–184 (2011).

5. Esteve-Altava, B., Marugán-Lobón, J., Botella, H., Bastir, M. & Rasskin-Gutman, D. Grist for Riedl’s mill: A network model perspective on the integration and modularity of the human skull. *J. Exp. Zoolog. B Mol. Dev. Evol.* **320,** 489–500 (2013).

6. Esteve-Altava, B., Marugán-Lobón, J., Botella, H. & Rasskin-Gutman, D. Structural constraints in the evolution of the tetrapod skull complexity: Williston’s Law revisited using network models. *Evol. Biol.* **40,** 209–219 (2013).

7. Rasskin-Gutman, D. & Esteve-Altava, B. Connecting the dots: Anatomical network analysis in morphological EvoDevo. *Biol. Theory* **9,** 178–193 (2014).

8. Esteve-Altava, B., Boughner, J. C., Diogo, R., Villmoare, B. A. & Rasskin-Gutman, D. Anatomical network analysis shows decoupling of modular lability and complexity in the evolution of the primate skull. *PLOS ONE* **10,** 1–17 (2015).

9. Adkins, R. M., Walton, A. H. & Honeycutt, R. L. Higher-level systematics of rodents and divergence time estimates based on two congruent nuclear genes. *Mol. Phylogenet. Evol.* **26,** 409–420 (2003).

10. Felsenstein, J. Phylogenies and the comparative method. *Am. Nat.* **125,** 1–15 (1985).

11. Revell, L. J. phytools: an R package for phylogenetic comparative biology (and other things). *Methods Ecol. Evol.* **3,** 217–223 (2012).

12. Pons, P. & Latapy, M. Computing communities in large networks using random walks. *J Graph Algorithms Appl* **10,** 191–218 (2006).

13. Newman, M. E. & Girvan, M. Finding and evaluating community structure in networks. *Phys. Rev. E* **69,** 1–16 (2004).

# 2- Details about results

# 2.a- MUSCULOSKELETAL NETWORKS

Bones and muscles are represented all as nodes. Links represent all types of contact among them.

**Tab. SI1-1.** Network parameters of heads.

|  | N | K | D | C | L | H |
| --- | --- | --- | --- | --- | --- | --- |
| Mus | 175 | 441 | 0.029 | 0.321 | 3.702 | 0.870 |
| Tupaia | 161 | 368 | 0.029 | 0.328 | 3.806 | 0.967 |
| Cynocephalus | 153 | 386 | 0.033 | 0.345 | 3.673 | 0.843 |
| Lemur | 151 | 355 | 0.031 | 0.350 | 3.781 | 0.905 |
| Propithecus | 148 | 329 | 0.030 | 0.360 | 3.707 | 1.023 |
| Loris | 149 | 350 | 0.032 | 0.328 | 3.657 | 0.910 |
| Nycticebus | 149 | 350 | 0.032 | 0.328 | 3.657 | 0.910 |
| Tarsius | 153 | 375 | 0.032 | 0.364 | 3.532 | 1.027 |
| Callithrix | 143 | 346 | 0.034 | 0.401 | 3.619 | 1.001 |
| Saimiri | 141 | 341 | 0.035 | 0.417 | 3.548 | 1.019 |
| Aotus | 145 | 338 | 0.032 | 0.445 | 3.613 | 1.035 |
| Pithecia | 143 | 320 | 0.032 | 0.381 | 3.693 | 1.077 |
| Colobus | 148 | 351 | 0.032 | 0.437 | 3.532 | 0.970 |
| Cercopithecus | 156 | 384 | 0.032 | 0.425 | 3.647 | 0.988 |
| Papio | 154 | 390 | 0.033 | 0.434 | 3.629 | 0.953 |
| Macaca | 154 | 367 | 0.031 | 0.412 | 3.642 | 1.023 |
| Hylobates | 155 | 357 | 0.030 | 0.401 | 3.653 | 1.047 |
| Pongo | 150 | 365 | 0.033 | 0.429 | 3.546 | 0.967 |
| Gorilla | 151 | 360 | 0.032 | 0.432 | 3.677 | 1.016 |
| Pan troglodytes | 154 | 358 | 0.030 | 0.433 | 3.564 | 1.038 |
| Pan paniscus | 152 | 357 | 0.031 | 0.447 | 3.527 | 1.030 |
| Homo | 157 | 352 | 0.029 | 0.412 | 3.589 | 1.067 |

**Fig. SI1-1** Phylogenetic mapping of the parameters. Colors indicate the range of values for each parameter as shown in the previous table. The states at internal nodes are estimated using maximum likelihood.

[1] "Number of nodes (N)"


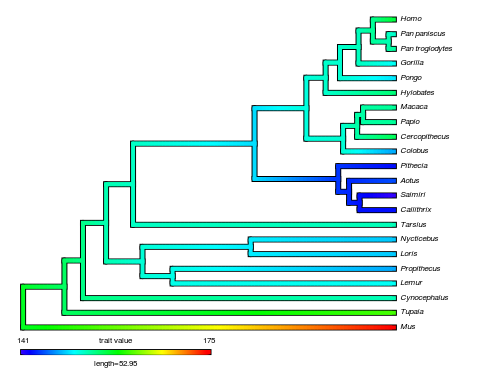


[1] "Number of connections (K)"
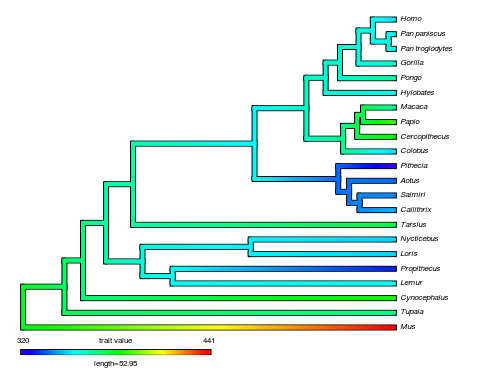


[1] "Density of connections (D)"
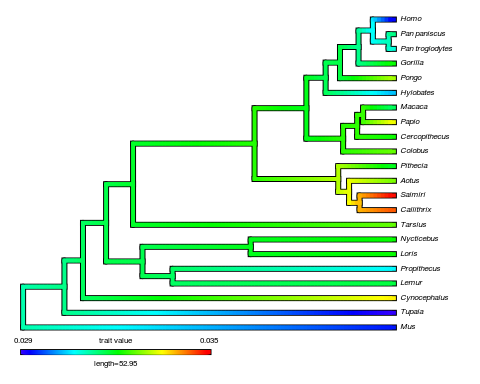


[1] "Clustering coefficient (C)"
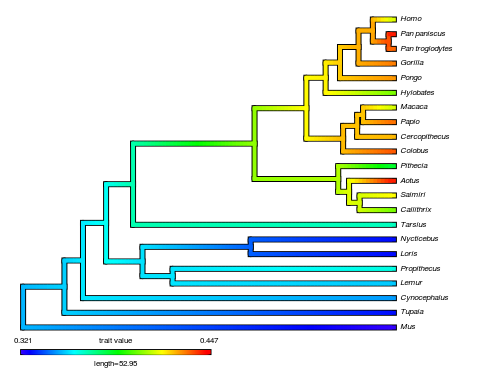


[1] "Shortest path length (L)"
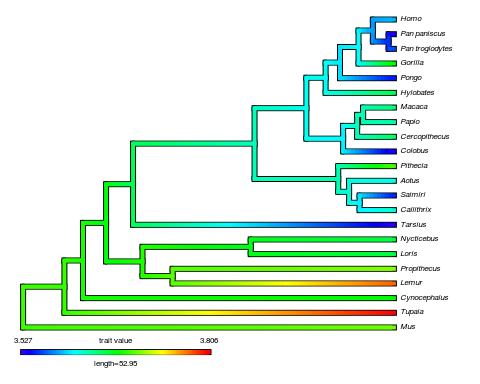


[1] "Heterogeneity (H)"


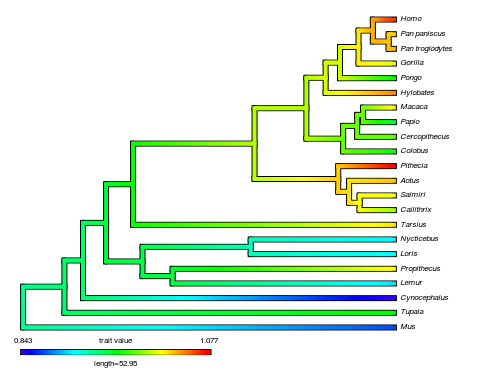


**Tab. SI1-2.** Best partitions identified using the random walk-trap algorithm.

|  | # Modules | Q value | Expected error |
| --- | --- | --- | --- |
| Mus | 20 | 0.5198708 | 0.0245026 |
| Tupaia | 19 | 0.5321177 | 0.0256901 |
| Cynocephalus | 15 | 0.4894360 | 0.0266594 |
| Lemur | 15 | 0.5540448 | 0.0251678 |
| Propithecus | 16 | 0.5371763 | 0.0269901 |
| Loris | 17 | 0.5142163 | 0.0273461 |
| Nycticebus | 17 | 0.5142163 | 0.0273461 |
| Tarsius | 16 | 0.5252836 | 0.0251856 |
| Callithrix | 12 | 0.5296368 | 0.0252986 |
| Saimiri | 13 | 0.5118979 | 0.0261995 |
| Aotus | 16 | 0.5290431 | 0.0268520 |
| Pithecia | 11 | 0.5033399 | 0.0251363 |
| Colobus | 17 | 0.5110998 | 0.0264261 |
| Cercopithecus | 15 | 0.5363702 | 0.0237661 |
| Papio | 14 | 0.5315812 | 0.0247583 |
| Macaca | 18 | 0.5062329 | 0.0257315 |
| Hylobates | 17 | 0.4839465 | 0.0276998 |
| Pongo | 12 | 0.5597486 | 0.0247454 |
| Gorilla | 17 | 0.4939044 | 0.0265606 |
| Pan troglodytes | 12 | 0.5320566 | 0.0254535 |
| Pan paniscus | 11 | 0.5363989 | 0.0254737 |
| Homo | 11 | 0.5268070 | 0.0265030 |

**Tab. SI1-3.** Musculoskeletal connectivity modules identified for the head of Mus.

| **ID** | **module** | **p-value** | **Elements** |
| --- | --- | --- | --- |
| 1 | Laryngeal and true vocal fold movement | 0 | arytenoid.cartilage.left, arytenoid.cartilage.right, laryngeal.alar.cartilage, cricoid.cartilage, constrictor.pharyngis.inferior.left, constrictor.pharyngis.inferior.right, cricothyroideus.left, cricothyroideus.right, thyroarytenoideus.left, thyroarytenoideus.right, cricoarytenoideus.lateralis.left, cricoarytenoideus.lateralis.right, arytenoideus, cricoarytenoideus.post..left, cricoarytenoideus.post..right, cricoarytenoideus.alaris.left, cricoarytenoideus.alaris.right |
| 2 | Neurocranium & facial, hyoid, pharyngeal and tongue muscles | 7.00E-05 | occipital, parietal.left, parietal.right, interparietal., squamosal.left, squamosal.right, mandible.left, mandible.right, hyoid.bone, thyroid.cartilage, occipitalis.left, occipitalis.right, auricularis.posterior.left, auricularis.posterior.right, mandibulo.auricularis.left, mandibulo.auricularis.right, orbito.temporo.auricularis.left, orbito.temporo.auricularis.right, stylohyoideus.left, stylohyoideus.right, jugulohyoideus.left, jugulohyoideus.right, constrictor.pharyngis.superior.left, constrictor.pharyngis.superior.right, geniohyoideus.left, geniohyoideus.right, genioglossus.left, genioglossus.right, thyrohyoideus.left, thyrohyoideus.right, depressor.septi.nasi.left, depressor.septi.nasi.right, nasalis..nasolabialis.profundus..left, nasalis..nasolabialis.profundus..right, depressor.rhinarii.left, depressor.rhinarii.right, levator.rhinarii.left, levator.rhinarii.right |
| 3 | Postcranial & infrahyoid muscles | 0.00054 | sternum, clavicle.left, clavicle.right, scapula.left, sternofacialis.left, sternofacialis.right, cleidooccipitalis.clavotrapezius.left, cleidooccipitalis.clavotrapezius.right, sternomastoideus.left, sternomastoideus.right, cleidomastoideus.left, cleidomastoideus.right, sternohyoideus.left, sternohyoideus.right, omohyoideus.left, omohyoideus.right, sternothryroideus.left, sternothryroideus.right |
| 4 | Platysma and zygomatic muscles | 0.00366 | platysma.myoides.left, platysma.myoides.right, platysma.cervicale..left, platysma.cervicale.right, zygomaticus.major.left, zygomaticus.major.right, zygomaticus.minor.left, zygomaticus.minor..right |
| 5 | Tympanic and basioccipital muscles | 0.02865 | tympanic.left, tympanic.right, basisphenoid, presphenoid, palatine.left, palatine.right, vomer, tensor.tympani.left, tensor.tympani.right, tensor.veli.palatini.left, tensor.veli.palatini.right, pterygoideus.lateralis.left, pterygoideus.lateralis.right, pterygoideus.medialis.left, pterygoideus.medialis.right |
| 6 | Right inner ear | 0.04318 | malleus.right, incus.right, stapes.right, stapedius.right |
| 7 | Left inner ear | 0.04318 | malleus.left, incus.left, stapes.left, stapedius.left |
| 8 | Right facial | 0.00209 | zygomatic.right, frontal.right, ethmoid., nasal.right, premaxilla.right, maxilla.right, lacrimal.right, inferior.nasal.concha.right, orbicularis.oculi.right, buccinatorius.right, levator.labii.sup.al.nasi..nasolabialis..right, dilatator.nasi.right, levator.labii.sup..max.naso.lab..right, levator.anguli.oris.facialis.right, orbicularis.oris.right, masseter.right, temporalis..2.bundles..right |
| 9 | Left facial | 0.00403 | zygomatic.left, frontal.left, nasal.left, premaxilla.left, maxilla.left, lacrimal.left, inferior.nasal.concha.left, orbicularis.oculi.left, buccinatorius.left, levator.labii.sup.al.nasi..nasolabialis..left, dilatator.nasi.left, levator.labii.sup..max.naso.lab..left, levator.anguli.oris.facialis.left, orbicularis.oris.left, masseter.left, temporalis..2.bundles..left |
| 10 | Pharyngeal muscles | 0.00017 | stylopharyngeus.left, stylopharyngeus.right, constrictor.pharyngis.medius.left, constrictor.pharyngis.medius.right, pterygopharyngeus.left, pterygopharyngeus.right, palatopharyngeus.left, palatopharyngeus.right, salpingopharyngeus.left, salpingopharyngeus.right |
| 11 | Right sphincter colli muscles | 0.65845 | sphincter.colli.superficialis.right, sphincter.colli.profundus.right |
| 12 | Left sphincter colli muscles | 0.65845 | sphincter.colli.superficialis.left, sphincter.colli.profundus.left |
| 13 | Dorsal postcranial | 0.00715 | scapula.right, vertebrae, acromiotrapezius.left, acromiotrapezius.right, spinotrapezius.left, spinotrapezius.right |
| 14 | Suprahyoid muscles | 0.05117 | mylohyoideus.left, mylohyoideus.right, digastricus.anterior.left, digastricus.anterior.right, digastricus.posterior.left, digastricus.posterior.right |
| 15 | Levator veli palatini | 1 | levator.veli.palatini.left, levator.veli.palatini.right |
| 16 | Left hyoglossus and styloglossus | 1 | hyoglossus.left, styloglossus.left |
| 17 | Right hyoglossus and styloglossus | 1 | hyoglossus.right, styloglossus.right |
| 18 | Intermandibularis | 1 | intermandibularis.anterior.left, intermandibularis.anterior.right |
| 19 | Ceratohyoideus | 1 | ceratohyoideus.left, ceratohyoideus.right |
| 20 | Interscutularis | 0.09697 | interscutularis.left, interscutularis.right |

**Tab. SI1-4.** Connectivity modules identified for the head of Tupaia.

| ID | module | p-value | Elements |
| --- | --- | --- | --- |
| 1 | Suprahyoid, pharyngeal, and tongue | 0 | hyoid.bone, mylohyoideus.left, mylohyoideus.right, digastricus.anterior.left, digastricus.anterior.right, stylohyoideus.left, stylohyoideus.right, digastricus.posterior.left, digastricus.posterior.right, jugulohyoideus.left, jugulohyoideus.right, stylopharyngeus.left, stylopharyngeus.right, ceratohyoideus.left, ceratohyoideus.right, constrictor.pharyngis.medius.left, constrictor.pharyngis.medius.right, palatopharyngeus.left, palatopharyngeus.right, salpingopharyngeus.left, salpingopharyngeus.right, geniohyoideus.left, geniohyoideus.right, genioglossus.left, genioglossus.right, hyoglossus.left, hyoglossus.right, thyrohyoideus.left, thyrohyoideus.right |
| 2 | Epicranial and ear movement | 0.05667 | occipitalis.left, occipitalis.right, frontalis.left, frontalis.right, auriculo.orbitalis.left, auriculo.orbitalis.right |
| 3 | Laryngeal and true vocal fold movement | 0 | thyroid.cartilage, arytenoid.left, arytenoid.right, cricoid.cartilage, constrictor.pharyngis.inferior.left, constrictor.pharyngis.inferior.right, cricothyroideus.left, cricothyroideus.right, thyroarytenoideus.left, thyroarytenoideus.right, cricoarytenoideus.lateralis.left, cricoarytenoideus.lateralis.right, arytenoideus.right, arytenoideus.right.1, cricoarytenoideus.posterior.left, cricoarytenoideus.posterior.right |
| 4 | Left facial | 5.00E-05 | ethmoid, frontal.left, lacrimal.left, maxilla.left, nasal.left, premaxilla.left, premaxilla.right, temporal.left, vomer, zygomatic.left, auricularis.posterior.left, zygomaticus.major.left, zygomaticus.minor.left, zygomatico.orbicularis.left, orbicularis.oculi.left, zygomatico.orbicularis.right.1, corrugator.supercilii.left, levator.labii.superioris.alaeque.nasi.left, buccinatorius.left, levator.labii.superioris.left, nasalis.left, levator.anguli.oris.facialis.left, orbicularis.oris.left, masseter.left, temporalis.main.body.left, styloglossus.left |
| 5 | Postcranial & infrahyoid muscles | 0.00243 | sternum, clavicle.left, clavicle.right, cleido.occipitalis.left, cleido.occipitalis.right, sternocleidomastoideus.left, sternocleidomastoideus.right, sternohyoideus.left, sternohyoideus.right, sternothryroideus.left, sternothryroideus.right |
| 6 | Neurocranium & facial, masticatory, and pharyngeal muscles | 0.00368 | occipital, palatine.left, palatine.right, parietal.left, parietal.right, sphenoid, temporal.right, mandible.left, mandible.right, auricularis.posterior.right, mandibulo.auricularis.left, mandibulo.auricularis.right, tensor.veli.palatini.left, tensor.veli.palatini.right, masseter.right, temporalis.main.body.right, pterygoideus.lateralis.left, pterygoideus.lateralis.right, pterygoideus.medialis.left, pterygoideus.medialis.right, constrictor.pharyngis.superior.left, constrictor.pharyngis.superior.right, levator.veli.palatini.left, levator.veli.palatini.right, styloglossus.right |
| 7 | Right facial | 0.0019 | frontal.right, lacrimal.right, maxilla.right, nasal.right, zygomatic.right, zygomaticus.major.right, zygomaticus.minor.right, zygomatico.orbicularis.right, orbicularis.oculi.right, zygomatico.orbicularis.left.1, corrugator.supercilii.right, levator.labii.superioris.alaeque.nasi.right, buccinatorius.right, levator.labii.superioris.right, nasalis.right, levator.anguli.oris.facialis.right, orbicularis.oris.right |
| 8 | Platisma and sphincter colli muscles | 0.00193 | platysma.cervicale.left, platysma.cervicale.right, platysma.myoides.left, platysma.myoides.right, sphincter.colli.superficialis.left, sphincter.colli.superficialis.right, sphincter.colli.profundus.left, sphincter.colli.profundus.right |
| 9 | Right malleus and incus | 1 | malleus.right, incus.right |
| 10 | Left malleus and incus | 1 | malleus.left, incus.left |
| 11 | Left scapula and omohyoideus | 0.40683 | scapula.left, omohyoideus.pars.superior.left, omohyoideus.pars.inferior.left |
| 12 | Right scapula and omohyoideus | 0.40683 | scapula.right, omohyoideus.pars.superior.right, omohyoideus.pars.inferior.right |
| 13 | Right stapes | 0.30854 | stapes.right, stapedius.right |
| 14 | Left stapes | 0.30854 | stapes.left, stapedius.left |
| 15 | Vertebrae and trapezius | 0.25249 | vertebrae, trapezius.left, trapezius.right |
| 16 | Mentalis | 1 | mentalis.left, mentalis.right |
| 17 | Intermandibularis | 1 | intermandibularis.anterior.left, intermandibularis.anterior.right |
| 18 | Left auricularis superior | 1 | auricularis.superior.left |
| 19 | Right auricularis superior | 1 | auricularis.superior.right |

**Tab. SI1-5.** Connectivity modules identified for the head of Cynocephalus.

| ID | module | p-value | Elements |
| --- | --- | --- | --- |
| 1 | Suprahyoid, pharyngeal, and tonuge | 0 | hyoid.bone, thyroid.cartilage, mylohyoideus.left, mylohyoideus.right, digastricus.anterior.left, digastricus.anterior.right, digastricus.posterior.left, digastricus.posterior.right, jugulohyoideus.left, jugulohyoideus.right, stylopharyngeus.left, stylopharyngeus.right, ceratorhyoideus.left, ceratohyoideus.right, constrictor.pharyngis.medius.left, constrictor.pharyngis.medius.right, pterygopharyngeus.left, pterygopharyngeus.right, palatopharyngeus.left, palatopharyngeus.right, salpingopharyngeus.left, salpingopharyngeus.right, geniohyoideus.left, geniohyoideus.right, genioglossus.left, genioglossus.right, hyoglossus.left, hyoglossus.right |
| 2 | Left incus and stapes | 0.07865 | incus.left, stapes.left, stapedius.left |
| 3 | Right incus and stapes | 0.07865 | incus.right, stapes.right, stapedius.right |
| 4 | Neurocranium & facial, masticatory, and pharyngeal muscles | 0 | ethmoid, maxilla.left, maxilla.right, occipital, palatine.left, palatine.right, parietal.left, parietal.right, premaxilla.left, premaxilla.right, sphenoid, temporal.left, temporal.right, vomer, mandible.left, mandible.right, platysma.cervicale.left, platysma.cervicale.right, platysma.myoides.left, platysma.myoides.right, occipitalis.left, occipitalis.right, auricularis.posterior.left, auricularis.posterior.right, sphincter.colli.profundus.left, sphincter.colli.profundus.right, tensor.veli.palatini.left, tensor.veli.palatini.right, masseter.left, masseter.right, temporalis.main.body.left, temporalis.main.body.right, pterygoideus.lateralis.left, pterygoideus.lateralis.right, pterygoideus.medialis.left, pterygoideus.medialis.right, constrictor.pharyngis.superior.left, constrictor.pharyngis.superior.right, levator.veli.palatini.left, levator.veli.palatini.right, styloglossus.left, styloglossus.right |
| 5 | Laryngeal and true vocal fold movement | 0 | arytenoid.left, arytenoid.right, cricoid.cartilage, constrictor.pharyngis.inferior.left, constrictor.pharyngis.inferior.right, cricothyroideus.left, cricothyroideus.right, thyroarytenoideus.left, thyroarytenoideus.right, cricoarytenoideus.lateralis.left, cricoarytenoideus.lateralis.right, arytenoideus.right, arytenoideus.right.1, cricoarytenoideus.posterior.left, cricoarytenoideus.posterior.right |
| 6 | Right facial | 0.00273 | frontal.right, lacrimal.right, nasal.right, zygomatic.right, zygomaticus.major.right, zygomaticus.minor.right, frontalis.right, auriculo.orbitalis.right, orbicularis.oculi.right, zygomatico.orbicularis.right, zygomatico.orbicularis.right, corrugator.supercilii.right, levator.labii.superioris.alaeque.nasi.right, buccinatorius.right, levator.labii.superioris.right, nasalis.right, levator.anguli.oris.facialis.right, orbicularis.oris.right |
| 7 | Left facial | 0.00273 | frontal.left, lacrimal.left, nasal.left, zygomatic.left, zygomaticus.major.left, zygomaticus.minor.left, frontalis.left, auriculo.orbitalis.left, orbicularis.oculi.left, zygomatico.orbicularis.left, zygomatico.orbicularis.left.1, corrugator.supercilii.left, levator.labii.superioris.alaeque.nasi.left, buccinatorius.left, levator.labii.superioris.left, nasalis.left, levator.anguli.oris.facialis.left, orbicularis.oris.left |
| 8 | Dorsal Postcranial | 0.00086 | scapula.left, scapula.right, vertebrae, acromiotrapezius.left, acromiotrapezius.right, spinotrapezius.left, spinotrapezius.right |
| 9 | Right malleus and tenso tympani | 0.65845 | malleus.right, tensor.tympani.right |
| 10 | Left malleus and tensor tympani | 0.65845 | malleus.left, tensor.tympani.left |
| 11 | Postcranial and infrahyoid muscles | 0.00218 | sternum, clavicle.left, clavicle.right, sternocleidomastoideus.left, sternocleidomastoideus.right, sternohyoideus.left, sternohyoideus.right, sternothryroideus.left, sternothryroideus.right |
| 12 | Mentalis | 1 | mentalis.left, mentalis.right |
| 13 | Palatoglossus | 1 | palatoglossus.left, palatoglossus.right |
| 14 | Let auricularis.superior | 1 | auricularis.superior.left |
| 15 | Right auricularis.superior | 1 | auricularis.superior.right |

**Tab. SI1-6.** Connectivity modules identified for the head of Lemur.

| **ID** | **module** | **p-value** | **Elements** |
| --- | --- | --- | --- |
|  |  |  |  |
| 1 | Neurocranium & facial, masticatory, and pharyngeal muscles | 1.00E-05 | occipital, palatine.left, palatine.right, parietal.left, parietal.right, sphenoid, temporal.left, temporal.right, mandible.left, occipitalis.left, occipitalis.right, mandibulo.auricularis.left, auricularis.posterior.left, auricularis.posterior.right, frontalis.left, frontalis.right, auriculo.orbitalis.left, auriculo.orbitalis.right, tensor.veli.palatini.left, tensor.veli.palatini.right, masseter.left, masseter.right, temporalis.main.body.left, temporalis.main.body.right, pterygoideus.lateralis.left, pterygoideus.medialis.left, pterygoideus.medialis.right, constrictor.pharyngis.superior.left, constrictor.pharyngis.superior.right, levator.veli.palatini.left, levator.veli.palatini.right |
| 2 | Postcranial | 0.01059 | clavicle.left, clavicle.right, scapula.left, scapula.right, vertebrae, trapezius.left, trapezius.right, omohyoideus.left, omohyoideus.right |
| 3 | Suprahyoid, pharyngeal and tongue | 1.00E-05 | hyoid.bone, mandible.right, mandibulo.auricularis.right, mylohyoideus.left, mylohyoideus.right, digastricus.anterior.left, digastricus.anterior.right, pterygoideus.lateralis.right, stylohyoideus.left, stylohyoideus.right, digastricus.posterior.left, digastricus.posterior.right, jugulohyoideus.left, jugulohyoideus.right, ceratohyoideus.left, ceratohyoideus.right, constrictor.pharyngis.medius.left, constrictor.pharyngis.medius.right, geniohyoideus.left, geniohyoideus.right, genioglossus.left, genioglossus.right, hyoglossus.left, hyoglossus.right, styloglossus.left, styloglossus.right, thyrohyoideus.left, thyrohyoideus.right |
| 4 | Postcranial & infrahyoid and facial muscles | 0.08921 | sternum, platysma.cervicale.left, platysma.cervicale.right, platysma.myoides.left, platysma.myoides.right, sphincter.colli.profundus.left, sphincter.colli.profundus.right, sternocleidomastoideus.left, sternocleidomastoideus.right, sternohyoideus.left, sternohyoideus.right, sternothryroideus.left, sternothryroideus.right |
| 5 | Right inner ear | 0.07865 | incus.right, stapes.right, stapedius.right |
| 6 | Left inner ear | 0.07865 | incus.left, stapes.left, stapedius.left |
| 7 | Left facial | 1.00E-05 | ethmoid, frontal.left, lacrimal.left, maxilla.left, nasal.left, premaxilla.left, premaxilla.right, vomer, zygomatic.left, zygomaticus.major.left, zygomaticus.minor.left, orbicularis.oculi.left, depressor.supercilii.left, corrugator.supercilii.left, levator.labii.superioris.alaeque.nasi.left, buccinatorius.left, levator.labii.superioris.left, nasalis.left, levator.anguli.oris.facialis.left, orbicularis.oris.left |
| 8 | Laryngeal movement | 0.00868 | thyroid.cartilage., stylopharyngeus.left, stylopharyngeus.right, constrictor.pharyngis.inferior.left, constrictor.pharyngis.inferior.right, cricothyroideus.left, cricothyroideus.right, palatopharyngeus.left, palatopharyngeus.right |
| 9 | True vocal fold movement | 0.00035 | arytenoid.left, arytenoid.right, cricoid.cartilage, thyroarytenoideus.left, thyroarytenoideus.right, cricoarytenoideus.lateralis.left, cricoarytenoideus.lateralis.right, arytenoideus.right, arytenoideus.right.1, cricoarytenoideus.posterior.left, cricoarytenoideus.posterior.right |
| 10 | Right facial | 0.00019 | frontal.right, lacrimal.right, maxilla.right, nasal.right, zygomatic.right, zygomaticus.major.right, zygomaticus..minor.right, orbicularis.oculi.right, depressor.supercilii.right, corrugator.supercilii.right, levator.labii.superioris.alaeque.nasi.right, buccinatorius.right, levator.labii.superioris.right, nasalis.right, levator.anguli.oris.facialis.right, orbicularis.oris.right |
| 11 | Right malleus and tensor tympani | 0.65845 | malleus.right, tensor.tympani.right |
| 12 | Left malleus and tensor tympani | 0.65845 | malleus.left, tensor.tympani.left |
| 13 | Mentalis | 1 | mentalis.left, mentalis.right |
| 14 | Left auricularis superior | 1 | auricularis.superior.left |
| 15 | Right auricularis superior | 1 | auricularis.superior.right |

**Tab. SI1-7.** Connectivity modules identified for the head of Propithecus.

| ID | module | p-value | Elements |
| --- | --- | --- | --- |
| 1 | Neurocranium & facial, masticatory, and pharyngeal muscles | 0 | occipital, palatine.left, palatine.right, parietal.left, parietal.right, sphenoid, temporal.left, temporal.right, mandible, platysma.cervicale.left, platysma.cervicale.right, platysma.myoides.left, platysma.myoides.right, occipitalis.left, occipitalis.right, mandibulo.auricularis.left, mandibulo.auricularis.right, auricularis.posterior.left, auricularis.posterior.right, mentalis.left, mentalis.right, tensor.veli.palatini.left, tensor.veli.palatini.right, masseter.left, masseter.right, temporalis.main.body.left, temporalis.main.body.right, pterygoideus.lateralis.left, pterygoideus.lateralis.right, pterygoideus.medialis.left, pterygoideus.medialis.right, constrictor.pharyngis.superior.left, constrictor.pharyngis.superior.right |
| 2 | Postcranial | 0.00323 | clavicle.left, clavicle.right, scapula.left, scapula.right, vertebrae, trapezius.left, trapezius.right, omohyoideus.left, omohyoideus.right |
| 3 | Suprahyoid, pharyngeal and tongue | 4.00E-05 | hyoid.bone, mylohyoideus.left, mylohyoideus.right, digastricus.anterior.left, digastricus.anterior.right, stylohyoideus.left, stylohyoideus.right, digastricus.posterior.left, digastricus.posterior.right, jugulohyoideus.right, jugulohyoideus.left, ceratohyoideus.left, ceratohyoideus.right, constrictor.pharyngis.medius.left, constrictor.pharyngis.medius.right, geniohyoideus.left, geniohyoideus.right, genioglossus.left, genioglossus.right, hyoglossus.left, hyoglossus.right, styloglossus.left, styloglossus.right |
| 4 | Postcranial & infrahyoid and pharyngeal muscles | 0.09858 | thyroid.cartilage, sternum, stylopharyngeus.left, stylopharyngeus.right, sternocleidomastoideus.left, sternocleidomastoideus.right, constrictor.pharyngis.inferior.left, constrictor.pharyngis.inferior.right, cricothyroideus.left, cricothyroideus.right, palatopharyngeus.left, palatopharyngeus.right, sternohyoideus.left, sternohyoideus.right, sternothryroideus.left, sternothryroideus.right, thyrohyoideus.left, thyrohyoideus.right |
| 5 | Right facial | 0.00012 | ethmoid, frontal.right, lacrimal.right, maxilla.right, nasal.right, premaxilla.left, premaxilla.right, vomer, zygomatic.right, zygomaticus..major.right, zygomaticus..minor.right, orbicularis.oculi.right, corrugator.supercilii.right, levator.labii.superioris.alaeque.nasi.right, buccinatorius.right, levator.labii.superioris.right, nasalis.right, levator.anguli.oris.facialis.right, orbicularis.oris.right |
| 6 | Right inner ear | 0.07865 | incus.right, stapes.right, stapedius.right |
| 7 | Left inner ear | 0.07865 | incus.left, stapes.left, stapedius.left |
| 8 | Left uper face and ear | 0.68132 | frontalis.left, auriculo.orbitalis.left, depressor.supercilii.left |
| 9 | Right upper face and ear | 0.68132 | frontalis.right, auriculo.orbitalis.right, depressor.supercilii.right |
| 10 | True vocal fold movement | 0.00035 | arytenoid.left, arytenoid.right, cricoid.cartilage, thyroarytenoideus.left, thyroarytenoideus.right, cricoarytenoideus.lateralis.left, cricoarytenoideus.lateralis.right, arytenoideus.right, arytenoideus.right.1, cricoarytenoideus.posterior.left, cricoarytenoideus.posterior.right |
| 11 | Left facial | 0.00118 | frontal.left, lacrimal.left, maxilla.left, nasal.left, zygomatic.left, zygomaticus.major.left, zygomaticus.minor.left, orbicularis.oculi.left, corrugator.supercilii.left, levator.labii.superioris.alaeque.nasi.left, buccinatorius.left, levator.labii.superioris.left, nasalis.left, levator.anguli.oris.facialis.left, orbicularis.oris.left |
| 12 | Right malleus and tensor tympani | 0.65845 | malleus.right, tensor.tympani.right |
| 13 | Left malleus and tensor tympani | 0.65845 | malleus.left, tensor.tympani.left |
| 14 | Levator veli palatini | 1 | levator.veli.palatini.left, levator.veli.palatini.right |
| 15 | Left auricularis superior | 1 | auricularis.superior.left |
| 16 | Right auricularis superior | 1 | auricularis.superior.right |

**Tab. SI1-8.** Connectivity modules identified for the head of Loris.

| ID | module | p-value | Elements |
| --- | --- | --- | --- |
|  |  |  |  |
| 1 | Suprahyoid, pharyngeal and tongue | 0.0004 | hyoid.bone, mylohyoideus.left, mylohyoideus.right, digastricus.anterior.right, stylohyoideus.right, digastricus.posterior.right, jugulohyoideus.left, jugulohyoideus.right, constrictor.pharyngis.medius.left, constrictor.pharyngis.medius.right, geniohyoideus.left, geniohyoideus.right, genioglossus.left, genioglossus.right, hyoglossus.left, hyoglossus.right, thyrohyoideus.left, thyrohyoideus.right |
| 2 | Neurocranium & facial, masticatory, and pharyngeal muscles | 0 | ethmoid, occipital, palatine.left, palatine.right, parietal.left, parietal.right, premaxilla.left, premaxilla.right, sphenoid, temporal.left, temporal.right, vomer, mandible.left, mandible.right, auricularis.posterior.left, auricularis.posterior.right, mandibulo.auricularis.left, mandibulo.auricularis.right, digastricus.anterior.left, tensor.veli.palatini.left, tensor.veli.palatini.right, masseter.left, masseter.right, temporalis.main.body.left, temporalis.main.body.right, pterygoideus.lateralis.left, pterygoideus.lateralis.right, pterygoideus.medialis.left, pterygoideus.medialis.right, stylohyoideus.left, digastricus.posterior.left, constrictor.pharyngis.superior.left, constrictor.pharyngis.superior.right, levator.veli.palatini.left, levator.veli.palatini.right, styloglossus.left, styloglossus.right |
| 3 | Epicranial& ear muscles and depressor supercilli | 0.063 | occipitalis.left, occipitalis.right, frontalis.left, frontalis.right, auriculo.orbitalis.left, auriculo.orbitalis.right, depressor.supercilii.left, depressor.supercilii.right |
| 4 | Right inner ear | 0.0787 | incus.right, stapes.right, stapedius.right |
| 5 | Left inner ear | 0.0787 | incus.left, stapes.left, stapedius.left |
| 6 | Postcranial & infrahyoid muscles | 0.0923 | sternum, platysma.cervicale.left, platysma.cervicale.right, platysma.myoides.left, platysma.myoides.right, sphincter.colli.profundus.left, sphincter.colli.profundus.right, sternohyoideus.left, sternohyoideus.right, sternothryroideus.left, sternothryroideus.right |
| 7 | True vocal fold movement | 0.0004 | arytenoid.left, arytenoid.right, cricoid.cartilage, thyroarytenoideus.left, thyroarytenoideus.right, cricoarytenoideus.lateralis.left, cricoarytenoideus.lateralis.right, arytenoideus.right, arytenoideus.right.1, cricoarytenoideus.posterior.left, cricoarytenoideus.posterior.right |
| 8 | Right postcranial & vertebrae | 0.0557 | clavicle.right, scapula.right, vertebrae, trapezius.right, sternocleidomastoideus.right, omohyoideus.right |
| 9 | Laryngeal movement | 0.0601 | thyroid.cartilage, stylopharyngeus.left, stylopharyngeus.right, constrictor.pharyngis.inferior.left, constrictor.pharyngis.inferior.right, cricothyroideus.left, cricothyroideus.right, palatopharyngeus.left, palatopharyngeus.right |
| 10 | Right facial | 0.0017 | frontal.right, lacrimal.right, maxilla.right, nasal.right, zygomatic.right, zygomaticus..major.right, zygomaticus..minor.right, orbicularis.oculi.right, corrugator.supercilii.right, levator.labii.superioris.alaeque.nasi.right, buccinatorius.right, levator.labii.superioris.right, nasalis.right, levator.anguli.oris.facialis.right, orbicularis.oris.right |
| 11 | Left facial | 0.0017 | frontal.left, lacrimal.left, maxilla.left, nasal.left, zygomatic.left, zygomaticus.major.left, zygomaticus.minor.left, orbicularis.oculi.left, corrugator.supercilii.left, levator.labii.superioris.alaeque.nasi.left, buccinatorius.left, levator.labii.superioris.left, nasalis.left, levator.anguli.oris.facialis.left, orbicularis.oris.left |
| 12 | Left postcranial | 0.0413 | clavicle.left, scapula.left, trapezius.left, sternocleidomastoideus.left, omohyoideus.left |
| 13 | Right malleus and tensor tympani | 0.6585 | malleus.right, tensor.tympani.right |
| 14 | Left malleus and tensor tympani | 0.6585 | malleus.left, tensor.tympani.left |
| 15 | Mentalis | 1 | mentalis.left, mentalis.right |
| 16 | Left auricularis superior | 1 | auricularis.superior.left |
| 17 | Right auricularis superior | 1 | auricularis.superior.right |

**Tab. SI1-9.** Connectivity modules identified for the head of Nycticebus.

| ID | module | p-value | Elements |
| --- | --- | --- | --- |
| 1 | Suprahyoid, pharyngeal and tongue | 0.00039 | hyoid.bone, mylohyoideus.left, mylohyoideus.right, digastricus.anterior.right, stylohyoideus.right, digastricus.posterior.right, jugulohyoideus.left, jugulohyoideus.right, constrictor.pharyngis.medius.left, constrictor.pharyngis.medius.right, geniohyoideus.left, geniohyoideus.right, genioglossus.left, genioglossus.right, hyoglossus.left, hyoglossus.right, thyrohyoideus.left, thyrohyoideus.right |
| 2 | Neurocranium & facial, masticatory, and pharyngeal muscles | 0 | ethmoid, occipital, palatine.left, palatine.right, parietal.left, parietal.right, premaxilla.left, premaxilla.right, sphenoid, temporal.left, temporal.right, vomer, mandible.left, mandible.right, auricularis.posterior.left, auricularis.posterior.right, mandibulo.auricularis.left, mandibulo.auricularis.right, digastricus.anterior.left, tensor.veli.palatini.left, tensor.veli.palatini.right, masseter.left, masseter.right, temporalis.main.body.left, temporalis.main.body.right, pterygoideus.lateralis.left, pterygoideus.lateralis.right, pterygoideus.medialis.left, pterygoideus.medialis.right, stylohyoideus.left, digastricus.posterior.left, constrictor.pharyngis.superior.left, constrictor.pharyngis.superior.right, levator.veli.palatini.left, levator.veli.palatini.right, styloglossus.left, styloglossus.right |
| 3 | Epicranial& ear muscles and depressor supercilli | 0.06302 | occipitalis.left, occipitalis.right, frontalis.left, frontalis.right, auriculo.orbitalis.left, auriculo.orbitalis.right, depressor.supercilii.left, depressor.supercilii.right |
| 4 | Right inner ear | 0.07865 | incus.right, stapes.right, stapedius.right |
| 5 | Left inner ear | 0.07865 | incus.left, stapes.left, stapedius.left |
| 6 | Postcranial & infrahyoid muscles | 0.09229 | sternum, platysma.cervicale.left, platysma.cervicale.right, platysma.myoides.left, platysma.myoides.right, sphincter.colli.profundus.left, sphincter.colli.profundus.right, sternohyoideus.left, sternohyoideus.right, sternothryroideus.left, sternothryroideus.right |
| 7 | True vocal fold movement | 0.00035 | arytenoid.left, arytenoid.right, cricoid.cartilage, thyroarytenoideus.left, thyroarytenoideus.right, cricoarytenoideus.lateralis.left, cricoarytenoideus.lateralis.right, arytenoideus.right, arytenoideus.right.1, cricoarytenoideus.posterior.left, cricoarytenoideus.posterior.right |
| 8 | Right postcranial & vertebrae | 0.05565 | clavicle.right, scapula.right, vertebrae, trapezius.right, sternocleidomastoideus.right, omohyoideus.right |
| 9 | Laryngeal movement | 0.0601 | thyroid.cartilage., stylopharyngeus.left, stylopharyngeus.right, constrictor.pharyngis.inferior.left, constrictor.pharyngis.inferior.right, cricothyroideus.left, cricothyroideus.right, palatopharyngeus.left, palatopharyngeus.right |
| 10 | Right facial | 0.00171 | frontal.right, lacrimal.right, maxilla.right, nasal.right, zygomatic.right, zygomaticus..major.right, zygomaticus.minor.right, orbicularis.oculi.right, corrugator.supercilii.right, levator.labii.superioris.alaeque.nasi.right, buccinatorius.right, levator.labii.superioris.right, nasalis.right, levator.anguli.oris.facialis.right, orbicularis.oris.right |
| 11 | Left facial | 0.00171 | frontal.left, lacrimal.left, maxilla.left, nasal.left, zygomatic.left, zygomaticus.major.left, zygomaticus.minor.left, orbicularis.oculi.left, corrugator.supercilii.left, levator.labii.superioris.alaeque.nasi.left, buccinatorius.left, levator.labii.superioris.left, nasalis.left, levator.anguli.oris.facialis.left, orbicularis.oris.left |
| 12 | Left postcranial | 0.04133 | clavicle.left, scapula.left, trapezius.left, sternocleidomastoideus.left, omohyoideus.left |
| 13 | Right malleus and tensor tympani | 0.65845 | malleus.right, tensor.tympani.right |
| 14 | Left malleus and tensor tympani | 0.65845 | malleus.left, tensor.tympani.left |
| 15 | Mentalis | 1 | mentalis.left, mentalis.right |
| 16 | Left auricularis superior | 1 | auricularis.superior.left |
| 17 | Right auricularis superior | 1 | auricularis.superior.right |

**Tab. SI1-10.** Connectivity modules identified for the head of Tarsius.

| ID | module | p-value | Elements |
| --- | --- | --- | --- |
| 1 | Supra- infrahyoid, and tongue | 0.00017 | hyoid.bone, mylohyoideus.left, mylohyoideus.right, digastricus.anterior.left, digastricus.anterior.right, stylohyoideus.left, stylohyoideus.right, digastricus.posterior.left, digastricus.posterior.right, ceratohyoideus.left, ceratohyoideus.right, constrictor.pharyngis.medius.left, constrictor.pharyngis.medius.right, geniohyoideus.left, geniohyoideus.right, hyoglossus.left, hyoglossus.right, sternohyoideus.left, sternohyoideus.right, sternothryroideus.left, sternothryroideus.right, thyrohyoideus.left, thyrohyoideus.right |
| 2 | Postcranial | 0.01805 | scapula.left, scapula.right, vertebrae, acromiotrapezius.left, acromiotrapezius.right, spinotrapezius.left, spinotrapezius.right, omohyoideus.left, omohyoideus.right |
| 3 | Neurocranium & facial, masticatory, and pharyngeal muscles | 4.00E-05 | ethmoid, lacrimal.right, occipital, palatine.left, palatine.right, parietal.left, parietal.right, sphenoid, temporal.left, temporal.right, vomer, occipitalis.left, occipitalis.right, auricularis.posterior.left, auricularis.posterior.right, zygomatico.auricularis.right, zygomatico.auricularis.left, tensor.veli.palatini.left, tensor.veli.palatini.right, masseter.left, masseter.right, temporalis.main.body.left, temporalis.main.body.right, pterygoideus.lateralis.left, pterygoideus.lateralis.right, pterygoideus.medialis.left, pterygoideus.medialis.right, constrictor.pharyngis.superior.left, constrictor.pharyngis.superior.right, musculus.uvulae.left, musculus.uvulae.right, levator.veli.palatini.left, levator.veli.palatini.right, styloglossus.left, styloglossus.right |
| 4 | Right incus and stapes | 0.07865 | incus.right, stapes.right, stapedius.right |
| 5 | Left incus and stapes | 0.07865 | incus.left, stapes.left, stapedius.left |
| 6 | Postcranial, mandible & and facial muscles | 0.00274 | mandible, sternum, clavicle.left, clavicle.right, platysma.cervicale.left, platysma.cervicale.right, platysma.myoides.left, platysma.myoides.right, sphincter.colli.profundus.left, sphincter.colli.profundus.right, sphincter.colli.profundus.left.1, sphincter.colli.profundus.right.1, mentalis.left, mentalis.right, sternocleidomastoideus.left, sternocleidomastoideus.right, genioglossus.left, genioglossus.right |
| 7 | Right facial | 0.00091 | maxilla.right, nasal.right, premaxilla.left, premaxilla.right, zygomatic.right, zygomaticus..major.right, zygomaticus..minor.right, frontalis.right, orbicularis.oculi.right, depressor.supercilii.right, corrugator.supercilii.right, levator.labii.superioris.alaeque.nasi.right, buccinatorius.right, levator.labii.superioris.right, nasalis.right, levator.anguli.oris.facialis.right, orbicularis.oris.right |
| 8 | True vocal fold movement | 0.00035 | arytenoid.left, arytenoid.right, cricoid.cartilage, thyroarytenoideus.left, thyroarytenoideus.right, cricoarytenoideus.lateralis.left, cricoarytenoideus.lateralis.right, arytenoideus.right, arytenoideus.right.1, cricoarytenoideus.posterior.left, cricoarytenoideus.posterior.right |
| 9 | Laryngeal | 0.00868 | thyroid.cartilage, stylopharyngeus.left, stylopharyngeus.right, constrictor.pharyngis.inferior.left, constrictor.pharyngis.inferior.right, cricothyroideus.left, cricothyroideus.right, palatopharyngeus.left, palatopharyngeus.right |
| 10 | Left facial | 0.00024 | frontal, lacrimal.left, maxilla.left, nasal.left, zygomatic.left, zygomaticus.major.left, zygomaticus.minor.left, frontalis.left, orbicularis.oculi.left, depressor.supercilii.left, corrugator.supercilii.left, levator.labii.superioris.alaeque.nasi.left, buccinatorius.left, levator.labii.superioris.left, nasalis.left, levator.anguli.oris.facialis.left, orbicularis.oris.left |
| 11 | Right malleus and tensor tympani | 0.65845 | malleus.right, tensor.tympani.right |
| 12 | Left malleus and tensor tympani | 0.65845 | malleus.left, tensor.tympani.left |
| 13 | Left auriculo-orbitalis | 1 | auriculo.orbitalis.left |
| 14 | Right auriculo-orbitalis | 1 | auriculo.orbitalis.right |
| 15 | Left auricularis-superior | 1 | auricularis.superior.left |
| 16 | Right auricularis-superior | 1 | auricularis.superior.right |

**Tab. SI1-11.** Connectivity modules identified for the head of Callithrix.

| ID | module | p-value | Elements |
| --- | --- | --- | --- |
| 1 | Neurocranium & facial, masticatory, and pharyngeal muscles | 0 | occipital, palatine.left, palatine.right, parietal.left, parietal.right, sphenoid, temporal.left, temporal.right, zygomatic.left, zygomatic.right, mandible, occipitalis.left, occipitalis.right, auricularis.posterior.left, auricularis.posterior.right, mentalis.left, mentalis.right, tensor.veli.palatini.left, tensor.veli.palatini.right, masseter.left, masseter.right, temporalis.main.body.left, temporalis.main.body.right, pterygoideus.lateralis.left, pterygoideus.lateralis.right, pterygoideus.medialis.left, pterygoideus.medialis.right, stylopharyngeus.left, stylopharyngeus.right, constrictor.pharyngis.superior.left, constrictor.pharyngis.superior.right, palatopharyngeus.left, palatopharyngeus.right, levator.veli.palatini.left, levator.veli.palatini.right, styloglossus.left, styloglossus.right |
| 2 | Supra-infrahyoid and tongue | 2.00E-05 | hyoid.bone, mylohyoideus.left, mylohyoideus.right, digastricus.anterior.left, digastricus.anterior.right, digastricus.posterior.left, digastricus.posterior.right, ceratohyoideus.left, ceratohyoideus.right, constrictor.pharyngis.medius.left, constrictor.pharyngis.medius.right, geniohyoideus.left, geniohyoideus.right, genioglossus.left, genioglossus.right, hyoglossus.left, hyoglossus.right, sternothryroideus.left, sternothryroideus.right, thyrohyoideus.left, thyrohyoideus.right |
| 3 | Laryngeal and true vocal fold movement | 0 | thyroid.cartilage, arytenoid.left, arytenoid.right, cricoid.cartilage, constrictor.pharyngis.inferior.left, constrictor.pharyngis.inferior.right, cricothyroideus.left, cricothyroideus.right, thyroarytenoideus.left, thyroarytenoideus.right, cricoarytenoideus.lateralis.left, cricoarytenoideus.lateralis.right, arytenoideus.right, arytenoideus.right.1, cricoarytenoideus.posterior.left, cricoarytenoideus.posterior.right |
| 4 | Postcranial | 0.0045 | scapula.left, scapula.right, vertebrae, trapezius.left, trapezius.right, omohyoideus.left, omohyoideus.right |
| 5 | Main facial | 0 | ethmoid, frontal, lacrimal.left, lacrimal.right, maxilla.left, maxilla.right, nasal.left, nasal.right, premaxilla.left, premaxilla.right, vomer, zygomaticus.left, zygomaticus.right, frontalis.left, frontalis.right, auriculo.orbitalis.left, auriculo.orbitalis.right, orbicularis.oculi.left, orbicularis.oculi.right, depressor.supercilii.left, depressor.supercilii.right, corrugator.supercilii.left, corrugator.supercilii.right, levator.labii.superioris.alaeque.nasi.left, levator.labii.superioris.alaeque.nasi.right, procerus.left, procerus.right, buccinatorius.left, buccinatorius.right, levator.labii.superioris.left, levator.labii.superioris.right, nasalis.left, nasalis.right, levator.anguli.oris.facialis.left, levator.anguli.oris.facialis.right, orbicularis.oris.left, orbicularis.oris.right |
| 6 | Left incus and stapes | 0.07865 | incus.left, stapes.left, stapedius.left |
| 7 | Right incus and stapes | 0.07865 | incus.right, stapes.right, stapedius.right |
| 8 | Postcranial & facial and infrahyoid | 8.00E-05 | sternum, clavicle.left, clavicle.right, platysma.cervicale.left, platysma.cervicale.right, platysma.myoides.left, platysma.myoides.right, spincter.colli.profundus.left, sphincter.colli.profundus.right, sternocleidomastoideus.left, sternocleidomastoideus.right, sternohyoideus.left, sternohyoideus.right |
| 9 | Right malleus and tensor tympani | 0.65845 | malleus.right, tensor.tympani.right |
| 10 | Left malleus and tensor tympani | 0.65845 | malleus.left, tensor.tympani.left |
| 11 | Left auricularis superior | 1 | auricularis.superior.left |
| 12 | Right auricularis superior | 1 | auricularis.superior.right |

**Tab. SI1-12.** Connectivity modules identified for the head of Saimiri.

| ID | module | p-value | Elements |
| --- | --- | --- | --- |
| 1 | Suprahyoid, pharyngeal and tongue | 0.00017 | hyoid.bone, mylohyoideus.left, mylohyoideus.right, digastricus.anterior.left, digastricus.anterior.right, digastricus.posterior.left, digastricus.posterior.right, ceratohyoideus.left, ceratohyoideus.right, constrictor.pharyngis.medius.left, constrictor.pharyngis.medius.right, geniohyoideus.left, geniohyoideus.right, genioglossus.left, genioglossus.right, hyoglossus.left, hyoglossus.right |
| 2 | Posctcranial | 0.00494 | clavicle.left, clavicle.right, scapula.left, scapula.right, vertebrae, trapezius.left, trapezius.right, omohyoideus.left, omohyoideus.right |
| 3 | Postcranial & facial and infrahyoid | 0.13741 | sternum, platysma.cervicale.left, platysma.cervicale.right, platysma.myoides.left, platysma.myoides.right, sphincter.colli.profundus.left, sphincter.colli.profundus.right, depressor.anguli.oris.left, depressor.anguli.oris.right, sternocleidomastoideus.left, sternocleidomastoideus.right, sternohyoideus.left, sternohyoideus.right, sternothryroideus.left, sternothryroideus.right |
| 4 | Neurocranium & facial, masticatory, and pharyngeal muscles | 1.00E-05 | occipital, palatine.left, palatine.right, parietal.left, parietal.right, sphenoid, temporal.left, temporal.right, mandible, occipitalis.left, occipitalis.right, auricularis.posterior.left, auricularis.posterior.right, mentalis.left, mentalis.right, tensor.veli.palatini.left, tensor.veli.palatini.right, masseter.left, masseter.right, temporalis.main.body.left, temporalis.main.body.right, pterygoideus.lateralis.left, pterygoideus.lateralis.right, pterygoideus.medialis.left, pterygoideus.medialis.right, constrictor.pharyngis.superior.left, constrictor.pharyngis.superior.right, musculus.uvulae.left, musculus.uvulae.right, levator.veli.palatini.left, levator.veli.palatini.right, styloglossus.left, styloglossus.right |
| 5 | Right inner ear | 0.07865 | incus.right, stapes.right, stapedius.right |
| 6 | Left inner ear | 0.07865 | incus.left, stapes.left, stapedius.left |
| 7 | Main facial | 0 | ethmoid, frontal, lacrimal.left, lacrimal.right, maxilla.left, maxilla.right, nasal.left, nasal.right, premaxilla.left, premaxilla.right, vomer, zygomatic.left, zygomatic.right, zygomaticus.left, zygomaticus.right, orbito.temporo.auricularis.left, orbito.temporo.auricularis.right, orbicularis.oculi.left, orbicularis.oculi.right, depressor.supercilii.left, depressor.supercilii.right, levator.labii.superioris.alaeque.nasi.left, levator.labii.superioris.alaeque.nasi.right, buccinatorius.left, buccinatorius.right, levator.labii.superioris.left, levator.labii.superioris.right, nasalis.left, nasalis.right, levator.anguli.oris.facialis.left, levator.anguli.oris.facialis.right, orbicularis.oris.left, orbicularis.oris.right |
| 8 | True vocal fold movement | 0.00864 | arytenoid.left, arytenoid.right, thyroarytenoideus.left, thyroarytenoideus.right, cricoarytenoideus.lateralis.left, cricoarytenoideus.lateralis.right, arytenoideus.right, arytenoideus.right.1, cricoarytenoideus.posterior.left, cricoarytenoideus.posterior.right |
| 9 | Laryngeal movement | 0.00524 | thyroid.cartilage, cricoid.cartilage, stylopharyngeus.left, stylopharyngeus.right, constrictor.pharyngis.inferior.left, constrictor.pharyngis.inferior.right, cricothyroideus.left, cricothyroideus.right, palatopharyngeus.left, palatopharyngeus.right, thyrohyoideus.left, thyrohyoideus.right |
| 10 | Left malleus and tensor tympani | 0.65845 | malleus.left, tensor.tympani.left |
| 11 | Right malleus and tensor tympani | 0.65845 | malleus.right, tensor.tympani.right |
| 12 | Left auricularis superior | 1 | auricularis.superior.left |
| 13 | Right auricularis superior | 1 | auricularis.superior.right |

**Tab. SI1-13.** Connectivity modules identified for the head of Aotus.

| ID | module | p-value | Elements |
| --- | --- | --- | --- |
| 1 | Postcranial & infrahyoid | 4.00E-05 | sternum, clavicle.left, clavicle.right, scapula.left, scapula.right, vertebrae, trapezius.left, trapezius.right, sternocleidomastoideus.left, sternocleidomastoideus.right, sternohyoideus.left, sternohyoideus.right, omohyoideus.left, omohyoideus.right, sternothryroideus.left, sternothryroideus.right |
| 2 | Suprahyoid, pharyngeal and tongue | 0 | hyoid.bone, mylohyoideus.left, mylohyoideus.right, digastricus.anterior.left, digastricus.anterior.right, stylohyoideus.left, stylohyoideus.right, digastricus.posterior.left, digastricus.posterior.right, ceratohyoideus.left, ceratohyoideus.right, constrictor.pharyngis.medius.left, constrictor.pharyngis.medius.right, geniohyoideus.left, geniohyoideus.right, genioglossus.left, genioglossus.right, hyoglossus.left, hyoglossus.right, thyrohyoideus.left, thyrohyoideus.right |
| 3 | Left facial | 0.0013 | maxilla.left, platysma.cervicale.left, platysma.cervicale.right, platysma.myoides.left, platysma.myoides.right, spincter.colli.profundus.left, sphincter.colli.profundus.right, frontalis.left, orbicularis.oculi.left, depressor.supercilii.left, levator.labii.superioris.alaeque.nasi.left, buccinatorius.left, levator.labii.superioris.left, nasalis.left, levator.anguli.oris.facialis.left, orbicularis.oris.left, depressor.anguli.oris.left, depressor.anguli.oris.right, mentalis.left |
| 4 | Left inner ear | 0.07865 | incus.left, stapes.left, stapedius.left |
| 5 | Right inner ear | 0.07865 | incus.right, stapes.right, stapedius.right |
| 6 | Right facial | 0.00021 | ethmoid, frontal, lacrimal.left, lacrimal.right, maxilla.right, nasal.left, nasal.right, premaxilla.left, premaxilla.right, frontalis.right, orbicularis.oculi.right, depressor.supercilii.right, corrugator.supercilii.left, corrugator.supercilii.right, levator.labii.superioris.alaeque.nasi.right, buccinatorius.right, levator.labii.superioris.right, nasalis.right, levator.anguli.oris.facialis.right, orbicularis.oris.right, mentalis.right |
| 7 | Neurocranium & facial, masticatory, and pharyngeal muscles | 0 | occipital, palatine.left, palatine.right, parietal.left, parietal.right, sphenoid, temporal.left, temporal.right, vomer, zygomatic.left, zygomatic.right, mandible, occipitalis.left, occipitalis.right, auricularis.posterior.left, auricularis.posterior.right, zygomaticus.left, zygomaticus.right, tensor.veli.palatini.left, tensor.veli.palatini.right, masseter.left, masseter.right, temporalis.main.body.left, temporalis.main.body.right, pterygoideus.lateralis.left, pterygoideus.lateralis.right, pterygoideus.medialis.left, pterygoideus.medialis.right, constrictor.pharyngis.superior.left, constrictor.pharyngis.superior.right, styloglossus.left, styloglossus.right |
| 8 | Laryngeal movement | 0.00094 | thyroid.cartilage, stylopharyngeus.left, stylopharyngeus.right, constrictor.pharyngis.inferior.left, constrictor.pharyngis.inferior.right, cricothyroideus.left, cricothyroideus.right, palatopharyngeus.left, palatopharyngeus.right |
| 9 | True vocal cord movement | 2.00E-04 | arytenoid.left, arytenoid.right, cricoid.cartilage, thyroarytenoideus.left, thyroarytenoideus.right, cricoarytenoideus.lateralis.left, cricoarytenoideus.lateralis.right, arytenoideus.right, arytenoideus.right.1, cricoarytenoideus.posterior.left, cricoarytenoideus.posterior.right |
| 10 | Left malleus and tensor tympani | 0.65845 | malleus.left, tensor.tympani.left |
| 11 | Right malleus and tensor tympani | 0.65845 | malleus.right, tensor.tympani.right |
| 12 | Levator veli palatini | 1 | levator.veli.palatini.left, levator.veli.palatini.right |
| 13 | Left auriculo-orbitalis | 1 | auriculo.orbitalis.left |
| 14 | Right auriculo-orbitalis | 1 | auriculo.orbitalis.right |
| 15 | Left auricularis superior | 1 | auricularis.superior.left |
| 16 | Right auricularis superior | 1 | auricularis.superior.right |

**Tab. SI1-14.** Connectivity modules identified for the head of Pithecia.

| ID | module | p-value | Elements |
| --- | --- | --- | --- |
| 1 | Postcranial & laryngeal movement | 0 | thyroid.cartilage, cricoid.cartilage, sternum, clavicle.left, clavicle.right, scapula.left, scapula.right, vertebrae, trapezius.left, trapezius.right, sternocleidomastoideus.left, sternocleidomastoideus.right, constrictor.pharyngis.inferior.left, constrictor.pharyngis.inferior.right, cricothyroideus.left, cricothyroideus.right, sternohyoideus.left, sternohyoideus.right, omohyoideus.left, omohyoideus.right, sternothryroideus.left, sternothryroideus.right, thyrohyoideus.left, thyrohyoideus.right |
| 2 | Main facial | 0 | ethmoid, frontal, lacrimal.left, lacrimal.right, maxilla.left, maxilla.right, nasal.left, nasal.right, premaxilla.left, premaxilla.right, platysma.cervicale.left, platysma.cervicale.right, platysma.myoides.left, platysma.myoides.right, occipitalis.left, occipitalis.right, zygomaticus.left, zygomaticus.right, frontalis.left, frontalis.right, auriculo.orbitalis.left, auriculo.orbitalis.right, orbicularis.oculi.left, orbicularis.oculi.right, depressor.supercilii.left, depressor.supercilii.right, corrugator.supercilii.left, corrugator.supercilii.right, levator.labii.superioris.alaeque.nasi.left, levator.labii.superioris.alaeque.nasi.right, buccinatorius.left, buccinatorius.right, levator.labii.superioris.left, levator.labii.superioris.right, nasalis.left, nasalis.right, levator.anguli.oris.facialis.left, levator.anguli.oris.facialis.right, orbicularis.oris.left, orbicularis.oris.right, depressor.anguli.oris.left, depressor.anguli.oris.right |
| 3 | Neurocranium, masticatory, suprahyoid, ear, and pharyngeal muscles | 0 | occipital, parietal.left, parietal.right, sphenoid, temporal.left, temporal.right, zygomatic.left, zygomatic.right, hyoid.bone, mandible, auricularis.posterior.left, auricularis.posterior.right, mentalis.left, mentalis.right, mylohyoideus.left, mylohyoideus.right, digastricus.anterior.left, digastricus.anterior.right, masseter.left, masseter.right, temporalis.main.body.left, temporalis.main.body.right, pterygoideus.lateralis.left, pterygoideus.lateralis.right, pterygoideus.medialis.left, pterygoideus.medialis.right, stylohyoideus.left, stylohyoideus.right, digastricus.posterior.left, digastricus.posterior.right, stylopharyngeus.left, stylopharyngeus.right, ceratohyoideus.left, ceratohyoideus.right, constrictor.pharyngis.medius.left, constrictor.pharyngis.medius.right, constrictor.pharyngis.superior.left, constrictor.pharyngis.superior.right, geniohyoideus.left, geniohyoideus.right, genioglossus.left, genioglossus.right, hyoglossus.left, hyoglossus.right, styloglossus.left, styloglossus.right |
| 4 | Palate muscles | 0.06242 | palatine.left, palatine.right, vomer, tensor.veli.palatini.left, tensor.veli.palatini.right, palatopharyngeus.left, palatopharyngeus.right, levator.veli.palatini.left, levator.veli.palatini.right |
| 5 | Left inner ear | 0.07865 | incus.left, stapes.left, stapedius.left |
| 6 | Right inner ear | 0.07865 | incus.right, stapes.right, stapedius.right |
| 7 | True vocal fold movement | 0.00209 | arytenoid.left, arytenoid.right, thyroarytenoideus.left, thyroarytenoideus.right, cricoarytenoideus.lateralis.left, cricoarytenoideus.lateralis.right, arytenoideus.right, arytenoideus.right.1, cricoarytenoideus.posterior.left, cricoarytenoideus.posterior.right |
| 8 | Left malleus and tensor tympani | 0.65845 | malleus.left, tensor.tympani.left |
| 9 | Right malleus and tensor tympani | 0.65845 | malleus.right, tensor.tympani.right |
| 10 | Left auricularis superior | 1 | auricularis.superior.left |
| 11 | Right auricularis superior | 1 | auricularis.superior.right |

**Tab. SI1-15.** Connectivity modules identified for the head of Colobus.

| ID |  | module | p-value | Elements |
| --- | --- | --- | --- | --- |
|  |  |  |  |  |
| 1 |  | Upper facial muscles | 0.00237 | frontalis.left, frontalis.right, auriculo.orbitalis.left, auriculo.orbitalis.right, orbicularis.oculi.left, orbicularis.oculi.right, depressor.supercilii.left, depressor.supercilii.right, corrugator.supercilii.left, corrugator.supercilii.right, procerus.left, procerus.right |
| 2 |  | Main facial | 0 | ethmoid, frontal, lacrimal.left, lacrimal.right, maxilla.left, maxilla.right, nasal.left, nasal.right, premaxilla.left, premaxilla.right, vomer, platsma.myoides.left, platsma.myoides.right, platysma.cervicale.left, platysma.cervicale.right, levator.labii.superioris.alaeque.nasi.left, levator.labii.superioris.alaeque.nasi.right, buccinatorius.left, buccinatorius.right, levator.labii.superioris.left, levator.labii.superioris.right, nasalis.left, nasalis.right, depressor.septi.nasi.left, depressor.septi.nasi.right, levator.anguli.oris.facialis.left, levator.anguli.oris.facialis.right, orbicularis.oris.left, orbicularis.oris.right, depressor.anguli.oris.left, depressor.anguli.oris.right |
| 3 |  | Laryngeal and true vocal fold movement | 2.00E-05 | thyroid.cartilage, arytenoid.left, arytenoid.right, cricoid.cartilage, constrictor.pharyngis.inferior.left, constrictor.pharyngis.inferior.right, cricothyroideus.left, cricothyroideus.right, thyroarytenoideus.left, thyroarytenoideus.right, cricoarytenoideus.lateralis.left, cricoarytenoideus.lateralis.right, arytenoideus, cricoarytenoideus.posterior.left, cricoarytenoideus.posterior.right |
| 4 |  | Postcranial | 0.0045 | clavicle.left, clavicle.right, scapula.left, scapula.right, vertebrae, trapezius.left, trapezius.right |
| 5 |  | Postcranial and infrahyoid | 0.12965 | sternum, sternocleidomastoideus.left, sternocleidomastoideus.right, hyoglossus.left, hyoglossus.right, styloglossus.left, styloglossus.right, sternohyoideus.left, sternohyoideus.right, sternothryroideus.left, sternothryroideus.right, thyrohyoideus.left, thyrohyoideus.right |
| 6 |  | Right inner ear | 0.07865 | incus.right, stapes.right, stapedius.right |
| 7 |  | Left inner ear | 0.07865 | incus.left, stapes.left, stapedius.left |
| 8 |  | Pharyngeal | 0.10515 | palatine.left, palatine.right, tensor.veli.palatini.left, tensor.veli.palatini.right, levator.veli.palatini.left, levator.veli.palatini.right, palatoglossus.left, palatoglossus.right |
| 9 |  | Suprahyoid and tongue | 0.00033 | hyoid.bone, mylohyoideus.left, mylohyoideus.right, digastricus.anterior.left, digastricus.anterior.right, stylohyoideus.left, stylohyoideus.right, digastricus.posterior.left, digastricus.posterior.right, geniohyoideus.left, geniohyoideus.right, genioglossus.left, genioglossus.right |
| 10 |  | Neurocranium & facial, masticatory, and pharyngeal muscles | 0.00025 | occipital, parietal.left, parietal.right, sphenoid, temporal.left, temporal.right, zygomatic.left, zygomatic.right, mandible, occipitalis.left, occipitalis.right, auricularis.posterior.left, auricularis.posterior.right, zygomaticus.major.left, zygomaticus.major.right, zygomaticus.minor.left, zygomaticus.minor.right, mentalis.left, mentalis.right, masseter.left, masseter.right, temporalis.main.body.left, temporalis.main.body.right, pterygoideus.lateralis.left, pterygoideus.lateralis.right, pterygoideus.medialis.left, pterygoideus.medialis.right, constrictor.pharyngis.superior.left, constrictor.pharyngis.superior.right |
| 11 |  | Left malleus and tensor tympani | 0.65845 | malleus.left, tensor.tympani.left |
| 12 |  | Right malleus and tynsor tympani | 0.65845 | malleus.right, tensor.tympani.right |
| 13 |  | Left longitudinal constrictors | 0.25249 | stylopharyngeus.left, palatopharyngeus.left, salpingopharyngeus.left |
| 14 |  | Right longitudinal constrictors | 0.25249 | stylopharyngeus.right, palatopharyngeus.right, salpingopharyngeus.right |
| 15 |  | Constrictor pharyngis medius | 1 | constrictor.pharyngis.medius.left, constrictor.pharyngis.medius.right |
| 16 |  | Left auricularis superior | 1 | auricularis.superior.left |
| 17 |  | Right auricularis superior | 1 | auricularis.superior.right |

**Tab. SI1-16.** Connectivity modules identified for the head of Cercopithecus.

| ID | module | p-value | Elements |
| --- | --- | --- | --- |
| 1 | Neurocranium & facial, masticatory, and pharyngeal muscles | 0 | occipital, palatine.left, palatine.right, parietal.left, parietal.right, sphenoid, temporal.left, temporal.right, zygomatic.left, zygomatic.right, mandible, vertebrae, occipitalis.left, occipitalis.right, auricularis.posterior.left, auricularis.posterior.right, zygomaticus.major.left, zygomaticus.major.right, zygomaticus.minor.left, mentalis.left, mentalis.right, tensor.veli.palatini.left, tensor.veli.palatini.right, masseter.left, masseter.right, temporalis.main.body.left, temporalis.main.body.right, pterygoideus.lateralis.left, pterygoideus.lateralis.right, pterygoideus.medialis.left, pterygoideus.medialis.right, constrictor.pharyngis.superior.left, constrictor.pharyngis.superior.right, levator.veli.palatini.left, levator.veli.palatini.right |
| 2 | Suprahyoid, pharyngeal and tongue | 0 | hyoid.bone, mylohyoideus.left, mylohyoideus.right, digastricus.anterior.left, digastricus.anterior.right, stylohyoideus.left, stylohyoideus.right, digastricus.posterior.left, digastricus.posterior.right, ceratohyoideus.left, ceratohyoideus.right, constrictor.pharyngis.medius.left, constrictor.pharyngis.medius.right, geniohyoideus.left, geniohyoideus.right, genioglossus.left, genioglossus.right, hyoglossus.left, hyoglossus.right, styloglossus.left, styloglossus.right, thyrohyoideus.left, thyrohyoideus.right |
| 3 | Postcranial and infrahyoid | 0.00069 | sternum, clavicle.left, clavicle.right, scapula.left, scapula.right, trapezius.left, trapezius.right, sternocleidomastoideus.left, sternocleidomastoideus.right, sternohyoideus.left, sternohyoideus.right, sternothryroideus.left, sternothryroideus.right |
| 4 | Laryngeal and true vocal fold movement | 0 | thyroid.cartilage, arytenoid.left, arytenoid.right, cricoid.cartilage, constrictor.pharyngis.inferior.left, constrictor.pharyngis.inferior.right, cricothyroideus.left, cricothyroideus.right, thyroarytenoideus.left, thyroarytenoideus.right, cricoarytenoideus.lateralis.left, cricoarytenoideus.lateralis.right, arytenoideus.right, cricoarytenoideus.posterior.left, cricoarytenoideus.posterior.right, ceratocricoideus.left, ceratocricoideus.right |
| 5 | Main facial | 0 | ethmoid, frontal, lacrimal.left, lacrimal.right, maxilla.left, maxilla.right, nasal.left, nasal.right, premaxilla.left, premaxilla.right, vomer, zygomaticus.minor.right, frontalis.left, frontalis.right, auriculo.orbitalis.left, auriculo.orbitalis.right, orbicularis.oculi.left, orbicularis.oculi.right, depressor.supercilii.left, depressor.supercilii.right, corrugator.supercilii.left, corrugator.supercilii.right, levator.labii.superioris.alaeque.nasi.left, levator.labii.superioris.alaeque.nasi.right, procerus.left, procerus.right, buccinatorius.left, buccinatorius.right, levator.labii.superioris.left, levator.labii.superioris.right, nasalis.left, nasalis.right, depressor.septi.nasi.left, depressor.septi.nasi.right, levator.anguli.oris.facialis.left, levator.anguli.oris.facialis.right, orbicularis.oris.left, orbicularis.oris.right |
| 6 | Right inner ear | 0.07865 | incus.right, stapes.right, stapedius.right |
| 7 | Left inner ear | 0.07865 | incus.left, stapes.left, stapedius.left |
| 8 | Lower facial | 0.44879 | platsma.myoides.left, platsma.myoides.right, platysma.cervicale.left, platysma.cervicale.right, sphincter.colli.profundus.left, sphincter.colli.profundus.right, depressor.labii.inferioris.left, depressor.labii.inferioris.right, depressor.anguli.oris.left, depressor.anguli.oris.right |
| 9 | Right malleus and tensor tympani | 0.65845 | malleus.right, tensor.tympani.right |
| 10 | Left malleus and tensor tympani | 0.65845 | malleus.left, tensor.tympani.left |
| 11 | Left longitudinal constrictors | 0.60187 | stylopharyngeus.left, palatopharyngeus.left, salpingopharyngeus.left |
| 12 | Right longitudinal constrictors | 0.60187 | stylopharyngeus.right, palatopharyngeus.right, salpingopharyngeus.right |
| 13 | Palatoglossus | 1 | palatoglossus.left, palatoglossus.right |
| 14 | Left Auricularis superior | 1 | auricularis.superior.left |
| 15 | Right Auricularis superior | 1 | auricularis.superior.right |

**Tab. SI1-17.** Connectivity modules identified for the head of Papio.

| ID | module | p-value | Elements |
| --- | --- | --- | --- |
| 1 | Supra-infrahyoid and tongue | 0 | hyoid.bone, sternum, mylohyoideus.left, mylohyoideus.right, digastricus.anterior.left, digastricus.anterior.right, stylohyoideus.left, stylohyoideus.right, digastricus.posterior.left, digastricus.posterior.right, ceratohyoideus.left, ceratohyoideus.right, sternocleidomastoideus.left, sternocleidomastoideus.right, constrictor.pharyngis.medius.left, constrictor.pharyngis.medius.right, geniohyoideus.left, geniohyoideus.right, genioglossus.left, genioglossus.right, hyoglossus.left, hyoglossus.right, styloglossus.left, styloglossus.right, sternohyoideus.left, sternohyoideus.right, sternothryroideus.left, sternothryroideus.right, thyrohyoideus.left, thyrohyoideus.right |
| 2 | Right facial | 0 | lacrimal.right, maxilla.right, nasal.right, premaxilla.right, platsma.myoides.left, platsma.myoides.right, platysma.cervicale.left, platysma.cervicale.right, zygomaticus.minor.right, frontalis.right, auriculo.orbitalis.right, orbicularis.oculi.right, depressor.supercilii.right, levator.labii.superioris.alaeque.nasi.right, procerus.right, buccinatorius.right, levator.labii.superioris.right, nasalis.right, depressor.septi.nasi.right, levator.anguli.oris.facialis.right, orbicularis.oris.right, depressor.labii.inferioris.left, depressor.labii.inferioris.right, depressor.anguli.oris.left, depressor.anguli.oris.right |
| 3 | Postcranial | 0.00494 | clavicle.left, clavicle.right, scapula.left, scapula.right, vertebrae, trapezius.left, trapezius.right, omohyoideus.left, omohyoideus.right |
| 4 | Longitudinal constrictors | 0.03445 | stylopharyngeus.left, stylopharyngeus.right, palatopharyngeus.left, palatopharyngeus.right, salpingopharyngeus.left, salpingopharyngeus.right |
| 5 | Left facial | 0.00029 | lacrimal.left, maxilla.left, nasal.left, premaxilla.left, zygomaticus.minor.left, frontalis.left, auriculo.orbitalis.left, orbicularis.oculi.left, depressor.supercilii.left, levator.labii.superioris.alaeque.nasi.left, procerus.left, buccinatorius.left, levator.labii.superioris.left, nasalis.left, depressor.septi.nasi.left, levator.anguli.oris.facialis.left, orbicularis.oris.left |
| 6 | Left inner ear | 0.07865 | incus.left, stapes.left, stapedius.left |
| 7 | Right inner ear | 0.07865 | incus.right, stapes.right, stapedius.right |
| 8 | Neurocranium & facial, masticatory, and pharyngeal muscles | 0 | ethmoid, frontal, occipital, palatine.left, palatine.right, parietal.left, parietal.right, sphenoid, temporal.left, temporal.right, vomer, zygomatic.left, zygomatic.right, mandible, occipitalis.left, occipitalis.right, auricularis.posterior.left, auricularis.posterior.right, zygomaticus.major.left, zygomaticus.major.right, corrugator.supercilii.left, corrugator.supercilii.right, tensor.veli.palatini.left, tensor.veli.palatini.right, masseter.left, masseter.right, temporalis.main.body.left, temporalis.main.body.right, pterygoideus.lateralis.left, pterygoideus.lateralis.right, pterygoideus.medialis.left, pterygoideus.medialis.right, constrictor.pharyngis.superior.left, constrictor.pharyngis.superior.right, levator.veli.palatini.left, levator.veli.palatini.right, palatoglossus.left, palatoglossus.right |
| 9 | Laryngeal and true vocal fold movement | 1.00E-05 | thyroid.cartilage, arytenoid.left, arytenoid.right, cricoid.cartilage, constrictor.pharyngis.inferior.left, constrictor.pharyngis.inferior.right, cricothyroideus.left, cricothyroideus.right, thyroarytenoideus.left, thyroarytenoideus.right, cricoarytenoideus.lateralis.left, cricoarytenoideus.lateralis.right, arytenoideus, cricoarytenoideus.posterior.left, cricoarytenoideus.posterior.right |
| 10 | Right malleus and tensor tympani | 0.65845 | malleus.right, tensor.tympani.right |
| 11 | Left malleus and tensor tympani | 0.65845 | malleus.left, tensor.tympani.left |
| 12 | Mentalis | 1 | mentalis.left, mentalis.right |
| 13 | Left auricularis superior | 1 | auricularis.superior.left |
| 14 | Right auricularis superior | 1 | auricularis.superior.right |

**Tab. SI1-18.** Connectivity modules identified for the head of Macaca.

| ID | module | p-value | Elements |
| --- | --- | --- | --- |
| 1 | Right facial | 1.00E-05 | ethmoid, frontal, lacrimal.left, lacrimal.right, maxilla.right, nasal.right, premaxilla.left, premaxilla.right, vomer, zygomatic.right, zygomaticus.major.right, zygomaticus.minor.right, frontalis.left, frontalis.right, orbicularis.oculi.right, depressor.supercilii.left, depressor.supercilii.right, corrugator.supercilii.left, corrugator.supercilii.right, levator.labii.superioris.alaeque.nasi.right, procerus.left, procerus.right, buccinatorius.right, levator.labii.superioris.right, nasalis.right, depressor.septi.nasi.right, levator.anguli.oris.facialis.right, orbicularis.oris.right |
| 2 | Laryngeal and true vocal fold movement | 1.00E-05 | thyroid.cartilage, arytenoid.left, arytenoid.right, cricoid.cartilage, constrictor.pharyngis.inferior.left, constrictor.pharyngis.inferior.right, cricothyroideus.left, cricothyroideus.right, thyroarytenoideus.left, thyroarytenoideus.right, cricoarytenoideus.lateralis.left, cricoarytenoideus.lateralis.right, arytenoideus, cricoarytenoideus.posterior.left, cricoarytenoideus.posterior.right |
| 3 | Left facial | 0.00184 | maxilla.left, nasal.left, platysma.myoides.left, platysma.myoides.right, platysma.cervicale.left, platysma.cervicale.right, levator.labii.superioris.alaeque.nasi.left, buccinatorius.left, levator.labii.superioris.left, nasalis.left, depressor.septi.nasi.left, levator.anguli.oris.facialis.left, orbicularis.oris.left, depressor.labii.inferioris.left, depressor.labii.inferioris.right, depressor.anguli.oris.left, depressor.anguli.oris.right |
| 4 | Right stapes and incus | 0.07865 | incus.right, stapes.right, stapedius.right |
| 5 | Left stapes and incus | 0.07865 | incus.left, stapes.left, stapedius.left |
| 6 | Postcranial, pharyngeal, tongue and infrahyoid | 0.29831 | sternum, stylopharyngeus.left, stylopharyngeus.right, sternocleidomastoideus.left, sternocleidomastoideus.right, palatopharyngeus.left, palatopharyngeus.right, hyoglossus.left, hyoglossus.right, styloglossus.left, styloglossus.right, sternohyoideus.left, sternohyoideus.right, sternothryroideus.left, sternothryroideus.right, thyrohyoideus.left, thyrohyoideus.right |
| 7 | Pharyngeal | 0.09876 | palatine.left, palatine.right, tensor.veli.palatini.left, tensor.veli.palatini.right, musculus.uvulae.left, musculus.uvulae.right, levator.veli.palatini.left, levator.veli.palatini.right, palatoglossus.left, palatoglossus.right |
| 8 | Neurocranium & facial, masticatory, and pharyngeal muscles | 0.00011 | occipital, parietal.left, parietal.right, sphenoid, temporal.left, temporal.right, zygomatic.left, mandible, occipitalis.left, occipitalis.right, auricularis.posterior.left, auricularis.posterior.right, zygomaticus.major.left, zygomaticus.minor.left, orbicularis.oculi.left, mentalis.left, mentalis.right, masseter.left, masseter.right, temporalis.main.body.left, temporalis.main.body.right, pterygoideus.lateralis.left, pterygoideus.lateralis.right, pterygoideus.medialis.left, pterygoideus.medialis.right, constrictor.pharyngis.superior.left, constrictor.pharyngis.superior.right |
| 9 | Suprahyoid and tongue | 4.00E-04 | hyoid.bone, mylohyoideus.left, mylohyoideus.right, digastricus.anterior.left, digastricus.anterior.right, stylohyoideus.left, stylohyoideus.right, digastricus.posterior.left, digastricus.posterior.right, ceratohyoideus.left, ceratohyoideus.right, geniohyoideus.left, geniohyoideus.right, genioglossus.left, genioglossus.right |
| 10 | Right postcranial | 0.2216 | clavicle.right, scapula.right, vertebrae, trapezius.right, omohyoideus.right |
| 11 | Left malleus and tensor tympani | 0.65845 | malleus.left, tensor.tympani.left |
| 12 | Right malleus and tensor tympani | 0.65845 | malleus.right, tensor.tympani.right |
| 13 | Left postcranial | 0.32659 | clavicle.left, scapula.left, trapezius.left, omohyoideus.left |
| 14 | Constrictor pharyngis medius | 1 | constrictor.pharyngis.medius.left, constrictor.pharyngis.medius.right |
| 15 | Left auriculo-orbitalis | 1 | auriculo.orbitalis.left |
| 16 | Right auriculo-orbitalis | 1 | auriculo.orbitalis.right |
| 17 | Left auricularis-superior | 1 | auricularis.superior.left |
| 18 | Right auricularis-superior | 1 | auricularis.superior.right |

**Tab. SI1-19.** Connectivity modules identified for the head of Hylobates.

| ID | module | p-value | Elements |
| --- | --- | --- | --- |
| 1 | Suprahyoid and tongue | 1.00E-05 | hyoid.bone, mylohyoideus.left, mylohyoideus.right, digastricus.anterior.left, digastricus.anterior.right, stylohyoideus.left, stylohyoideus.right, digastricus.posterior.left, digastricus.posterior.right, constrictor.pharyngis.medius.left, constrictor.pharyngis.medius.right, geniohyoideus.left, geniohyoideus.right, genioglossus.left, genioglossus.right, hyoglossus.left, hyoglossus.right, styloglossus.left, styloglossus.right |
| 2 | Laryngeal and vocal fold movement | 0 | thyroid.cartilage, arytenoid.left, arytenoid.right, cricoid.cartilage, constrictor.pharyngis.inferior.left, constrictor.pharyngis.inferior.right, cricothyroideus.left, cricothyroideus.right, thyroideus.transversus, thyroarytenoideus.left, thyroarytenoideus.right, cricoarytenoideus.lateralis.left, cricoarytenoideus.lateralis.right, arytenoideus, cricoarytenoideus.posterior.left, cricoarytenoideus.posterior.right, thyrohyoideus.left, thyrohyoideus.right |
| 3 | Neurocranium & facial, masticatory and pharyngeal muscles | 0 | maxilla.left, maxilla.right, occipital, palatine.left, palatine.right, parietal.left, parietal.right, premaxilla.left, premaxilla.right, sphenoid, temporal.left, temporal.right, vomer, zygomatic.left, malleus.left, malleus.right, mandible, tensor.tympani.left, tensor.tympani.right, occipitalis.left, occipitalis.right, auricularis.posterior.left, auricularis.posterior.right, tensor.veli.palatini.left, tensor.veli.palatini.right, masseter.left, masseter.right, temporalis.main.body.left, temporalis.main.body.right, pterygoideus.lateralis.left, pterygoideus.lateralis.right, pterygoideus.medialis.left, pterygoideus.medialis.right, constrictor.pharyngis.superior.left, constrictor.pharyngis.superior.right, pterygopharyngeus.left, pterygopharyngeus.right, levator.veli.palatini.left, levator.veli.palatini.right, palatoglossus.left, palatoglossus.right |
| 4 | Left facial | 0.0215 | platysma.myoides.left, platysma.myoides.right, platysma.cervicale.left, platysma.cervicale.right, zygomaticus.major.left, zygomaticus.minor.left, levator.labii.superioris.alaeque.nasi.left, buccinatorius.left, levator.labii.superioris.left, nasalis.left, depressor.septi.nasi.left, levator.anguli.oris.facialis.left, orbicularis.oris.left, depressor.labii.inferioris.left, depressor.labii.inferioris.right, depressor.anguli.oris.left, depressor.anguli.oris.right |
| 5 | Left upper facial | 0.2216 | frontalis.left, auriculo.orbitalis.left, orbicularis.oculi.left, depressor.supercilii.left, corrugator.supercilii.left |
| 6 | Right upper facial | 0.2216 | frontalis.right, auriculo.orbitalis.right, orbicularis.oculi.right, depressor.supercilii.right, corrugator.supercilii.right |
| 7 | Right facial | 0.00246 | ethmoid, frontal, lacrimal.left, lacrimal.right, nasal.left, nasal.right, zygomatic.right, zygomaticus.major.right, zygomaticus.minor.right, levator.labii.superioris.alaeque.nasi.right, procerus.left, procerus.right, buccinatorius.right, levator.labii.superioris.right, nasalis.right, depressor.septi.nasi.right, levator.anguli.oris.facialis.right, orbicularis.oris.right |
| 8 | Right postcranial | 0.09798 | clavicle.right, scapula.right, vertebrae, trapezius.right, omohyoideus.right |
| 9 | Right inner ear | 0.15085 | incus.right, stapes.right, stapedius.right |
| 10 | Left inner ear | 0.15085 | incus.left, stapes.left, stapedius.left |
| 11 | Left postcranial | 0.08602 | clavicle.left, scapula.left, trapezius.left, omohyoideus.left |
| 12 | Postcranial & infrahyoid | 0.83196 | sternum, sternocleidomastoideus.left, sternocleidomastoideus.right, sternohyoideus.left, sternohyoideus.right, sternothryroideus.left, sternothryroideus.right |
| 13 | Right longitudinal constrictors | 0.09835 | stylopharyngeus.right, palatopharyngeus.right, salpingopharyngeus.right |
| 14 | Left longitudinal constrictors | 0.09835 | stylopharyngeus.left, palatopharyngeus.left, salpingopharyngeus.left |
| 15 | Mentalis | 1 | mentalis.left, mentalis.right |
| 16 | Left auricularis superior | 1 | auricularis.superior.left |
| 17 | Right auricularis superior | 1 | auricularis.superior.right |

**Tab. SI1-20.** Connectivity modules identified for the head of Pongo.

| ID | module | p-value | Elements |
| --- | --- | --- | --- |
| 1 | Neurocranium & facial, masticatory, and pharyngeal muscles | 0 | malleus.left, malleus.right, occipital, palatine.left, palatine.right, parietal.left, parietal.right, sphenoid, temporal.left, temporal.right, vomer, mandible, tensor.tympani.left, tensor.tympani.right, mentalis.left, mentalis.right, tensor.veli.palatini.left, tensor.veli.palatini.right, masseter.left, masseter.right, temporalis.main.body.left, temporalis.main.body.right, pterygoideus.lateralis.pars.superior.left, pterygoideus.lateralis.pars.superior.right, pterygoideus.medialis.left, pterygoideus.medialis.right, stylolaryngeus.left, stylolaryngeus.right, digastricus.posterior.left, digastricus.posterior.right, constrictor.pharyngis.superior.left, constrictor.pharyngis.superior.right, levator.veli.palatini.left, levator.veli.palatini.right, palatoglossus.left, palatoglossus.right |
| 2 | Right facial | 0.00109 | maxilla.right, premaxilla.left, premaxilla.right, zygomatic.right, zygomaticus.major.right, zygomaticus.minor.right, orbicularis.oculi.right, depressor.supercilii.right, levator.labii.superioris.alaeque.nasi.right, procerus.right, buccinatorius.right, levator.labii.superioris.right, nasalis.right, depressor.septi.nasi.right, levator.anguli.oris.facialis.right, orbicularis.oris.right |
| 3 | Suprahyoid and tongue | 0.00157 | hyoid.bone, mylohyoideus.left, mylohyoideus.right, stylohyoideus.left, stylohyoideus.right, geniohyoideus.left, geniohyoideus.right, genioglossus.left, genioglossus.right, hyoglossus.left, hyoglossus.right, styloglossus.left, styloglossus.right |
| 4 | Left facial | 1.00E-05 | ethmoid, frontal, lacrimal.left, lacrimal.right, maxilla.left, nasal.left, nasal.right, zygomatic.left, zygomaticus.major.left, zygomaticus.minor.left, orbicularis.oculi.left, depressor.supercilii.left, corrugator.supercilii.left, corrugator.supercilii.right, levator.labii.superioris.alaeque.nasi.left, procerus.left, buccinatorius.left, levator.labii.superioris.left, nasalis.left, depressor.septi.nasi.left, levator.anguli.oris.facialis.left, orbicularis.oris.left |
| 5 | Laryngeal movement | 0.00113 | thyroid.cartilage, cricoid.cartilage, stylopharyngeus.left, constrictor.pharyngis.medius.left, constrictor.pharyngis.medius.right, constrictor.pharyngis.inferior.left, constrictor.pharyngis.inferior.right, cricothyroideus.left, cricothyroideus.right, palatopharyngeus.left, salpingopharyngeus.left, thyrohyoideus.left, thyrohyoideus.right |
| 6 | Postcranial | 3.00E-05 | sternum, clavicle.left, clavicle.right, scapula.left, scapula.right, vertebrae, platysma.myoides.left, playtsma.myoides.right, platysma.cervicale.left, platysma.cervicale.right, depressor.labii.inferioris.left, depressor.labii.inferioris.right, depressor.anguli.oris.left, depressor.anguli.oris.right, trapezius.left, trapezius.right, sternocleidomastoideus.left, sternocleidomastoideus.right, sternohyoideus.left, sternohyoideus.right, omohyoideus.left, omohyoideus.right, sternothryroideus.left, sternothryroideus.right |
| 7 | True vocal fold movement | 0.00565 | arytenoid.left, arytenoid.right, thyroarytenoideus.left, thyroarytenoideus.right, cricoarytenoideus.lateralis.left, cricoarytenoideus.lateralis.right, arytenoideus, cricoarytenoideus.posterior.left, cricoarytenoideus.posterior.right |
| 8 | Right epicranial and ear movement | 0.02575 | occipitalis.right, frontalis.right, auriculo.orbitalis.right, auricularis.superior.right |
| 9 | Left epicranial and ear movement | 0.02575 | occipitalis.left, frontalis.left, auriculo.orbitalis.left, auricularis.superior.left |
| 10 | Right inner ear | 0.15085 | incus.right, stapes.right, stapedius.right |
| 11 | Left inner ear | 0.15085 | incus.left, stapes.left, stapedius.left |
| 12 | Right longitudinal constrictors | 0.25249 | stylopharyngeus.right, palatopharyngeus.right, salpingopharyngeus.right |

**Tab. SI1-21.** Connectivity modules identified for the head of Gorilla.

| ID | module | p-value | Elements |
| --- | --- | --- | --- |
| 1 | Suprahyoid and tongue | 0.00041 | hyoid.bone, mylohyoideus.left, mylohyoideus.right, digastricus.anterior.left, digastricus.anterior.right, stylohyoideus.left, stylohyoideus.right, digastricus.posterior.left, digastricus.posterior.right, geniohyoideus.left, geniohyoideus.right, genioglossus.left, genioglossus.right, hyoglossus.left, hyoglossus.right, styloglossus.left, styloglossus.right, thyrohyoideus.left, thyrohyoideus.right |
| 2 | Left facial | 3.00E-05 | ethmoid, frontal, lacrimal.left, maxilla.left, nasal.left, premaxilla.left, premaxilla.right, platysma.myoides.left, platysma.myoides.right, zygomaticus.major.left, zygomaticus.minor.left, frontalis.left, auriculo.orbitalis.left, orbicularis.oculi.left, depressor.supercilii.left, corrugator.supercilii.left, corrugator.supercilii.right, levator.labii.superioris.alaeque.nasi.left, procerus.left, procerus.right, buccinatorius.left, levator.labii.superioris.left, nasalis.left, depressor.septi.nasi.left, levator.anguli.oris.facialis.left, orbicularis.oris.left, depressor.labii.inferioris.left, depressor.labii.inferioris.right, depressor.anguli.oris.left, depressor.anguli.oris.right |
| 3 | Laryngeal movement | 0.00186 | thyroid.cartilage, cricoid.cartilage, stylopharyngeus.left, stylopharyngeus.right, constrictor.pharyngis.inferior.left, constrictor.pharyngis.inferior.right, cricothyroideus.left, cricothyroideus.right, palatopharyngeus.left, palatopharyngeus.right, salpingopharyngeus.left, salpingopharyngeus.right |
| 4 | Pharygeal | 0.0142 | palatine.left, palatine.right, sphenoid, vomer, tensor.veli.palatini.left, tensor.veli.palatini.right, levator.veli.palatini.left, levator.veli.palatini.right, palatoglossus.left, palatoglossus.right |
| 5 | Left inner ear | 0.60393 | malleus.left, incus.left, tensor.tympani.left |
| 6 | Right inner ear | 0.60393 | malleus.right, incus.right, tensor.tympani.right |
| 7 | Right facial | 0.0036 | lacrimal.right, maxilla.right, nasal.right, zygomaticus.major.right, zygomaticus.minor.right, frontalis.right, auriculo.orbitalis.right, orbicularis.oculi.right, depressor.supercilii.right, levator.labii.superioris.alaeque.nasi.right, buccinatorius.right, levator.labii.superioris.right, nasalis.right, depressor.septi.nasi.right, levator.anguli.oris.facialis.right, orbicularis.oris.right |
| 8 | True vocal fold movement | 0.00864 | arytenoid.left, arytenoid.right, thyroarytenoideus.left, thyroarytenoideus.right, cricoarytenoideus.lateralis.left, cricoarytenoideus.lateralis.right, arytenoideus.transversus, arytenoideus.obliquus, cricoarytenoideus.posterior.left, cricoarytenoideus.posterior.right |
| 9 | Neurocranium& facial, masticatory, and pharyngeal muscles | 0.00198 | occipital, parietal.left, parietal.right, temporal.left, temporal.right, zygomatic.left, zygomatic.right, mandible, occipitalis.left, occipitalis.right, auricularis.posterior.left, auricularis.posterior.right, mentalis.left, mentalis.right, masseter.left, masseter.right, temporalis.main.body.left, temporalis.main.body.right, pterygoideus.lateralis.pars.superior.left, pterygoideus.lateralis.pars.superior.right, pterygoideus.medialis.left, pterygoideus.medialis.right, constrictor.pharyngis.superior.left, constrictor.pharyngis.superior.right |
| 10 | Left postcranial | 0.13666 | clavicle.left, scapula.left, vertebrae, trapezius.left, omohyoideus.single.belly.left |
| 11 | Postcranial & infrahyoid | 0.55664 | sternum, sternocleidomastoideus.left, sternocleidomastoideus.right, sternohyoideus.left, sternohyoideus.right, sternothryroideus.left, sternothryroideus.right |
| 12 | Right postcranial | 0.17645 | clavicle.right, scapula.right, trapezius.right, omohyoideus.single.belly.right |
| 13 | Right stapes and stapedius | 0.30854 | stapes.right, stapedius.right |
| 14 | Left stapes and stapedius | 0.30854 | stapes.left, stapedius.left |
| 15 | Constrictor pharyngis medius | 1 | constrictor.pharyngis.medius.left, constrictor.pharyngis.medius.right |
| 16 | Left auricularis superior | 1 | auricularis.superior.left |
| 17 | Right auricularis superior | 1 | auricularis.superior.right |

**Tab. SI1-22.** Connectivity modules identified for the head of Pan troglodytes.

| **ID** | **module** | **p-value** | **Elements** |
| --- | --- | --- | --- |
| 1 | **Postcranial** | 0.06289 | clavicle.left, clavicle.right, scapula.right, vertebrae, trapezius.left, trapezius.right, sternocleidomastoideus.left, sternocleidomastoideus.right, omohyoideus.pars.superior.right, omohyoideus.pars.inferior.right |
| 2 | **Supra-infrahyoid and tongue** | 0 | hyoid.bone, sternum, mylohyoideus.left, mylohyoideus.right, digastricus.anterior.left, digastricus.anterior.right, stylohyoideus.left, stylohyoideus.right, digastricus.posterior.left, digastricus.posterior.right, constrictor.pharyngis.medius.left, constrictor.pharyngis.medius.right, geniohyoideus.left, geniohyoideus.right, genioglossus.left, genioglossus.right, hyoglossus.left, hyoglossus.right, styloglossus.left, styloglossus.right, sternohyoideus.left, sternohyoideus.right, sternothryroideus.left, sternothryroideus.right, thyrohyoideus.left, thyrohyoideus.right |
| 3 | **Left facial** | 6.32E-03 | premaxilla.left, premaxilla.right, maxilla.left, zygomaticus.major.left, zygomaticus.minor.left, auriculo.orbitalis.left, depressor.supercilii.left, levator.labii.superioris.alaeque.nasi.left, levator.labii.superioris.left, nasalis.left, depressor.septi.nasi.left, levator.anguli.oris.facialis.left, orbicularis.oris.left |
| 4 | **Right facial** | 0 | frontal, ethmoidal, nasal.left, nasal.right, maxilla.right, lacrimal.left, lacrimal.right, zygomaticus.major.right, zygomaticus.minor.right, frontalis.left, frontalis.right, auriculo.orbitalis.right, orbicularis.oculi.left, orbicularis.oculi.right, depressor.supercilii.right, corrugator.supercilii.left, corrugator.supercilii.right, levator.labii.superioris.alaeque.nasi.right, procerus.left, procerus.right, buccinatorius.right, levator.labii.superioris.right, nasalis.right, depressor.septi.nasi.right, levator.anguli.oris.facialis.right, orbicularis.oris.right |
| 5 | **Pharyngeal** | 0.00682 | palatine.left, palatine.right, vomer, tensor.veli.palatini.left, tensor.veli.palatini.right, stylopharyngeus.left, stylopharyngeus.right, palatopharyngeus.left, palatopharyngeus.right, levator.veli.palatini.left, levator.veli.palatini.right, salpingopharyngeus.left, salpingopharyngeus.right, palatoglossus.left, palatoglossus.right |
| 6 | **Laryngeal and true vocal fold movement** | 1.00E-05 | thyroid.cartilage, arytenoid.left, arytenoid.right, cricoid.cartilage, constrictor.pharyngis.inferior.left, constrictor.pharyngis.inferior.right, cricothyroideus.left, cricothyroideus.right, thyroarytenoideus.left, thyroarytenoideus.right, cricoarytenoideus.lateralis.left, cricoarytenoideus.lateralis.right, arytenoideus, cricoarytenoideus.posterior.left, cricoarytenoideus.posterior.right |
| 7 | **Neurocranium & facial, masticatory muscles and pharyngeal muscles** | 0 | occipital, parietal.left, parietal.right, temporal.left, temporal.right, sphenoid, zygomatic.left, zygomatic.right, malleus.left, malleus.right, mandible, tensor.tympani.left, tensor.tympani.right, platysma.myoides.left, platysma.myoides.right, occipitalis.left, occipitalis.right, auricularis.posterior.left, auricularis.posterior.right, buccinatorius.left, depressor.labii.inferioris.left, depressor.labii.inferioris.right, depressor.anguli.oris.left, depressor.anguli.oris.right, mentalis.left, mentalis.right, masseter.left, masseter.right, temporalis.main.body.left, temporalis.main.body.right, pterygoideus.lateralis.pars.superior.left, pterygoideus.lateralis.pars.superior.right, pterygoideus.lateralis.pars.inferior.left, pterygoideus.lateralis.pars.inferior.right, pterygoideus.medialis.left, pterygoideus.medialis.right, constrictor.pharyngis.superior.left, constrictor.pharyngis.superior.right |
| 8 | **Left scapula and omohyoideus** | 0.40683 | scapula.left, omohyoideus.pars.superior.left, omohyoideus.pars.inferior.left |
| 9 | **Right inner ear** | 0.15085 | incus.right, stapes.right, stapedius.right |
| 10 | **Left inner ear** | 0.15085 | incus.left, stapes.left, stapedius.left |
| 11 | **Left auricularis superior** | 1 | auricularis.superior.left |
| 12 | **Right auricularis superior** | 1 | auricularis.superior.right |

**Tab. SI1-23.** Connectivity modules identified for the head of Pan paniscus.

| **ID** | **module** | **p-value** | **Elements** |
| --- | --- | --- | --- |
| 1 | **Supra-infrahyoid and tongue** | Su | hyoid.bone, sternum, mylohyoideus.left, mylohyoideus.right, digastricus.anterior.left, digastricus.anterior.right, stylohyoideus.left, stylohyoideus.right, digastricus.posterior.left, digastricus.posterior.right, constrictor.pharyngis.medius.left, constrictor.pharyngis.medius.right, geniohyoideus.left, geniohyoideus.right, genioglossus.left, genioglossus.right, hyoglossus.left, hyoglossus.right, styloglossus.left, styloglossus.right, sternohyoideus.left, sternohyoideus.right, sternothryroideus.left, sternothryroideus.right, thyrohyoideus.left, thyrohyoideus.right |
| 2 | **Left facial** | 6.32E-03 | premaxilla.left, premaxilla.right, maxilla.left, zygomaticus.major.left, zygomaticus.minor.left, auriculo.orbitalis.left, depressor.supercilii.left, levator.labii.superioris.alaeque.nasi.left, levator.labii.superioris.left, nasalis.left, depressor.septi.nasi.left, levator.anguli.oris.facialis.left, orbicularis.oris.left, buccinatorius left |
| 3 | **Right facial** | 0.00E+00 | frontal, ethmoidal, nasal.left, nasal.right, maxilla.right, lacrimal.left, lacrimal.right, zygomaticus.major.right, zygomaticus.minor.right, frontalis.left, frontalis.right, auriculo.orbitalis.right, orbicularis.oculi.left, orbicularis.oculi.right, depressor.supercilii.right, corrugator.supercilii.left, corrugator.supercilii.right, levator.labii.superioris.alaeque.nasi.right, procerus.left, procerus.right, levator.labii.superioris.right, nasalis.right, depressor.septi.nasi.right, levator.anguli.oris.facialis.right, orbicularis.oris.right, buccinatorius.right |
| 4 | **Pharyngeal** | 6.82E-03 | palatine.left, palatine.right, vomer, tensor.veli.palatini.left, tensor.veli.palatini.right, stylopharyngeus.left, stylopharyngeus.right, palatopharyngeus.left, palatopharyngeus.right, levator.veli.palatini.left, levator.veli.palatini.right, salpingopharyngeus.left, salpingopharyngeus.right, palatoglossus.left, palatoglossus.right |
| 5 | **Postcranial** | 0.01152 | clavicle.left, clavicle.right, scapula.left, scapula.right, vertebrae, trapezius.left, trapezius.right, sternocleidomastoideus.left, sternocleidomastoideus.right, omohyoideus.left, omohyoideus.right |
| 6 | **Laryngeal and true vocal fold movement** | 1.00E-05 | thyroid.cartilage, arytenoid.left, arytenoid.right, cricoid.cartilage, constrictor.pharyngis.inferior.left, constrictor.pharyngis.inferior.right, cricothyroideus.left, cricothyroideus.right, thyroarytenoideus.left, thyroarytenoideus.right, cricoarytenoideus.lateralis.left, cricoarytenoideus.lateralis.right, arytenoideus, cricoarytenoideus.posterior.left, cricoarytenoideus.posterior.right |
| 7 | **Neurocranium & facial, masticatory and pharyngeal muscles** | 0 | occipital, parietal.left, parietal.right, temporal.left, temporal.right, sphenoid, zygomatic.left, zygomatic.right, malleus.left, malleus.right, mandible, tensor.tympani.left, tensor.tympani.right, platysma.myoides.left, platysma.myoides.right, occipitalis.left, occipitalis.right, auricularis.posterior.left, auricularis.posterior.right, depressor.labii.inferioris.left, depressor.labii.inferioris.right, depressor.anguli.oris.left, depressor.anguli.oris.right, mentalis.left, mentalis right, masseter.left, masseter.right, temporalis.main.body.left, temporalis.main.body.right, pterygoideus.lateralis.pars.superior.left, pterygoideus.lateralis.pars.superior.right, pterygoideus.lateralis.pars.inferior.left, pterygoideus.lateralis.pars.inferior.right, pterygoideus.medialis.left, pterygoideus.medialis.right, constrictor.pharyngis.superior.left, constrictor.pharyngis.superior.right |
| 8 | **Right inner ear** | 0.15085 | incus.right, stapes.right, stapedius.right |
| 9 | **Left inner ear** | 0.15085 | incus.left, stapes.left, stapedius.left |
| 10 | **Left auricularis superior** | 1 | auricularis.superior.left |
| 11 | **Right auricularis superior** | 1 | auricularis.superior.right |

**Tab. SI1-24** Connectivity modules identified for the head of Homo.

| **ID** | **module** | **p-value** | **Elements** |
| --- | --- | --- | --- |
| 1 | **Postcranial** | 8.70E-04 | clavicle.left, clavicle.right, scapula.left, scapula.right, vertebrae, trapezius.left, trapezius.right, omohyoideus.pars.superior.left, omohyoideus.pars.superior.right, omohyoideus.pars.inferior.left, omohyoideus.pars.inferior.right |
| 2 | **Left facial** | 0.00E+00 | zygomatic.left, frontal, nasal.left, nasal.right, maxilla.left, lacrimal.left, platysma.myoides.left, risorius.left, zygomaticus.major.left, zygomaticus.minor.left, frontalis.left, frontalis.right, orbicularis.oculi.left, depressor.supercilii.left, corrugator.supercilii.left, corrugator.supercilii.right, levator.labii.superioris.alaeque.nasi.left, procerus.left, procerus.right, buccinatorius.left, levator.labii.superioris.left, nasalis.left, depressor.septi.nasi.left, levator.anguli.oris.facialis.left, orbicularis.oris.left, depressor.labii.inferioris.left, depressor.anguli.oris.left |
| 3 | **Supra-infrahyoid and tongue** | 4.00E-05 | hyoid.bone, sternum, mylohyoideus.left, mylohyoideus.right, digastricus.anterior.left, digastricus.anterior.right, stylohyoideus.left, stylohyoideus.right, digastricus.posterior.left, digastricus.posterior.right, constrictor.pharyngis.medius.left, constrictor.pharyngis.medius.right, geniohyoideus.left, geniohyoideus.right, genioglossus.left, genioglossus.right, hyoglossus.left, hyoglossus.right, styloglossus.left, styloglossus.right, sternohyoideus.left, sternohyoideus.right, sternothryroideus.left, sternothryroideus.right |
| 4 | **Neurocranium & facial, masticatory and pharyngeal muscles** | 0.00E+00 | occipital, parietal.left, parietal.right, temporal.left, temporal.right, sphenoid, ethmoidal, palatine.left, palatine.right, vomer, malleus.left, malleus.right, mandible, tensor.tympani.left, tensor.tympani.right, occipitalis.left, occipitalis.right, auticularis.posterior.left, auticularis.posterior.right, mentalis.left, mentalis.right, tensor.veli.palatini.left, tensor.veli.palatini.right, masseter.left, masseter.right, temporalis.main.body.left, temporalis.main.body.right, pterygoideus.lateralis.pars.superior.left, pterygoideus.lateralis.pars.superior.right, pterygoideus.lateralis.pars.inferior.left, pterygoideus.lateralis.pars.inferior.right, pterygoideus.medialis.left, pterygoideus.medialis.right, sternocleidomastoideus.left, sternocleidomastoideus.right, constrictor.pharyngis.superior.left, constrictor.pharyngis.superior.right, levator.veli.palatini.left, levator.veli.palatini.right, palatoglossus.left, palatoglossus.right |
| 5 | **Laryngeal movement** | 8.30E-04 | thyroid.cartilage, cricoid.cartilage, stylopharyngeus.left, stylopharyngeus.right, constrictor.pharyngis.inferior.left, constrictor.pharyngis.inferior.right, cricothyroideus.left, cricothyroideus.right, palatopharyngeus.left, palatopharyngeus.right, salpingopharyngeus.left, salpingopharyngeus.right, thyrohyoideus.left, thyrohyoideus.right |
| 6 | **Right facial** | 2.90E-04 | zygomatic.right, maxilla.right, lacrimal.right, platysma.myoides.right, risorius.right, zygomaticus.major.right, zygomaticus.minor.right, orbicularis.oculi.right, depressor.supercilii.right, levator.labii.superioris.alaeque.nasi.right, buccinatorius.right, levator.labii.superioris.right, nasalis.right, depressor.septi.nasi.right, levator.anguli.oris.facialis.right, orbicularis.oris.right, depressor.labii.inferioris.right, depressor.anguli.oris.right |
| 7 | **True vocal fold movement** | 8.64E-03 | arytenoid.left, arytenoid.right, thyroarytenoideus.left, thyroarytenoideus.right, cricoarytenoideus.lateralis.left, cricoarytenoideus.lateralis.right, arytenoideus.transversus, arytenoideus.obliquus, cricoarytenoideus.posterior.left, cricoarytenoideus.posterior.right |
| 8 | **Right ear facial muscles** | 2.97E-02 | temporoparietalis.right, auticularis.anterior.right, auricularis.superior.right |
| 9 | **Left ear facial muscles** | 0.02967 | temporoparietalis.left, auticularis.anterior.left, auricularis.superior.left |
| 10 | **Right inner ear** | 0.15085 | incus.right, stapes.right, stapedius.right |
| 11 | **Left inner ear** | 0.15085 | incus.left, stapes.left, stapedius.left |

# 2.b- SKELETAL NETWORKS

Nodes represent only the bones. Links represent physical articulations among bones.

**Tab. SI1-25.** Network parameters of heads.

|  | N | K | D | C | L | H |
| --- | --- | --- | --- | --- | --- | --- |
| Mus | 48 | 95 | 0.084 | 0.403 | 2.261 | 0.789 |
| Tupaia | 41 | 83 | 0.101 | 0.358 | 2.738 | 0.763 |
| Cynocephalus | 41 | 79 | 0.096 | 0.334 | 2.759 | 0.744 |
| Lemur | 41 | 75 | 0.091 | 0.254 | 2.809 | 0.693 |
| Propithecus | 40 | 72 | 0.092 | 0.271 | 2.787 | 0.711 |
| Loris | 41 | 81 | 0.099 | 0.312 | 2.788 | 0.720 |
| Nycticebus | 41 | 81 | 0.099 | 0.312 | 2.788 | 0.720 |
| Tarsius | 39 | 75 | 0.101 | 0.379 | 2.656 | 0.817 |
| Callithrix | 39 | 75 | 0.101 | 0.341 | 2.758 | 0.756 |
| Saimiri | 39 | 74 | 0.100 | 0.369 | 2.737 | 0.793 |
| Aotus | 39 | 73 | 0.099 | 0.317 | 2.775 | 0.762 |
| Pithecia | 39 | 73 | 0.099 | 0.346 | 2.806 | 0.735 |
| Colobus | 39 | 73 | 0.099 | 0.330 | 2.763 | 0.765 |
| Cercopithecus | 39 | 73 | 0.099 | 0.330 | 2.763 | 0.765 |
| Papio | 39 | 75 | 0.101 | 0.388 | 2.754 | 0.823 |
| Macaca | 39 | 73 | 0.099 | 0.330 | 2.763 | 0.765 |
| Hylobates | 39 | 76 | 0.103 | 0.384 | 2.732 | 0.800 |
| Pongo | 39 | 69 | 0.093 | 0.320 | 2.801 | 0.794 |
| Gorilla | 39 | 74 | 0.100 | 0.373 | 2.654 | 0.811 |
| Pan troglodytes | 39 | 70 | 0.094 | 0.341 | 2.813 | 0.813 |
| Pan paniscus | 39 | 70 | 0.094 | 0.341 | 2.813 | 0.813 |
| Homo | 37 | 72 | 0.108 | 0.403 | 2.711 | 0.803 |

**Fig. SI1-2.** Phylogenetic mapping of the parameters. Colors indicate the range of values for each parameter as shown in the previous table. The states at internal nodes is estimated using maximum likelihood.

[1] "Number of nodes (N)"


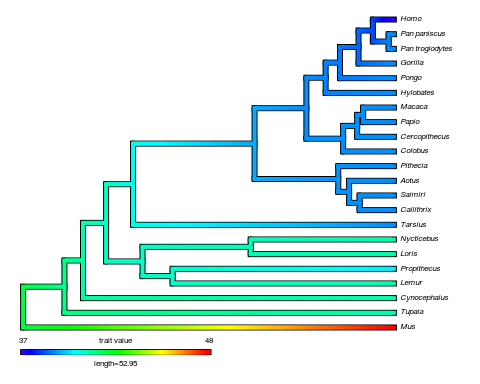


[1] "Number of connections (K)"
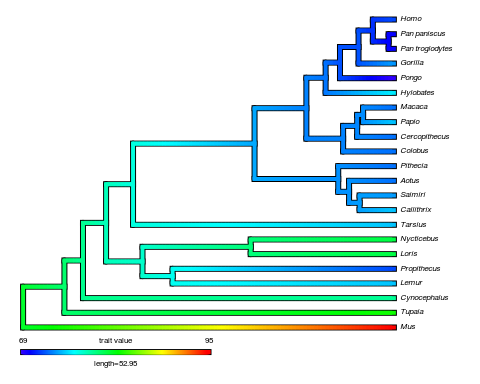


[1] "Density of connections (D)"
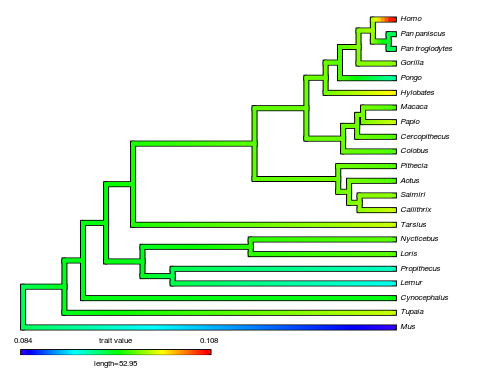


[1] "Clustering coefficient (C)"
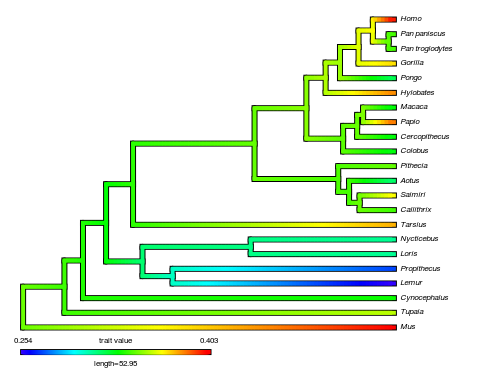


[1] "Shortest path length (L)"
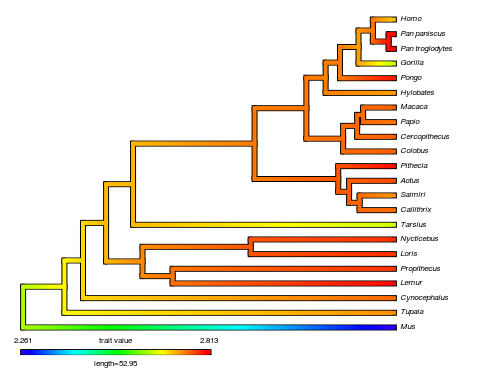


[1] "Heterogeneity (H)"


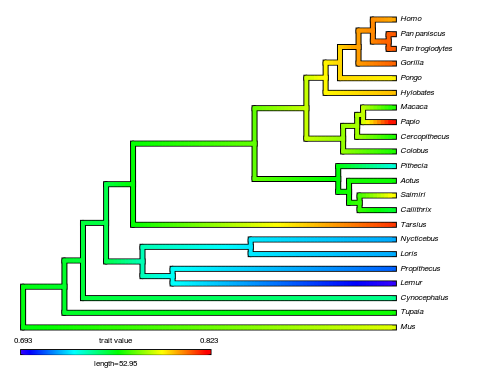


**Tab. SI1-26.** Best partitions identified using the random walk-trap algorithm.

|  | # Modules | Q value | Expected error |
| --- | --- | --- | --- |
| Mus | 13 | 0.4682549 | 0.0493727 |
| Tupaia | 8 | 0.4248803 | 0.0551747 |
| Cynocephalus | 8 | 0.3910431 | 0.0607965 |
| Lemur | 8 | 0.4175111 | 0.0616024 |
| Propithecus | 8 | 0.4154128 | 0.0633541 |
| Loris | 8 | 0.3991008 | 0.0599774 |
| Nycticebus | 8 | 0.3991008 | 0.0599774 |
| Tarsius | 10 | 0.3807111 | 0.0638113 |
| Callithrix | 8 | 0.4495111 | 0.0571549 |
| Saimiri | 8 | 0.4375457 | 0.0589241 |
| Aotus | 8 | 0.4500844 | 0.0582827 |
| Pithecia | 8 | 0.4625633 | 0.0569825 |
| Colobus | 8 | 0.3964158 | 0.0635173 |
| Cercopithecus | 8 | 0.3964158 | 0.0635173 |
| Papio | 8 | 0.3908444 | 0.0627909 |
| Macaca | 8 | 0.3964158 | 0.0635173 |
| Hylobates | 8 | 0.3880713 | 0.0624375 |
| Pongo | 11 | 0.4500105 | 0.0611727 |
| Gorilla | 8 | 0.3936267 | 0.0631506 |
| Pan troglodytes | 9 | 0.4092857 | 0.0651701 |
| Pan paniscus | 9 | 0.4092857 | 0.0651701 |
| Homo | 8 | 0.3992091 | 0.0638915 |

**Tab. SI 1-27.** Connectivity modules identified for the head of *Mus*.

| ID | p-value | Elements |
| --- | --- | --- |
| 1 | 7e-04 | occipital, parietal.left, parietal.right, interparietal., squamosal.left, squamosal.right, tympanic.left, tympanic.right, basisphenoid, zygomatic.left |
| 2 | 0.00885 | thyroid.cartilage, arytenoid.cartilage.left, arytenoid.cartilage.right, cricoid.cartilage |
| 3 | 0.00015 | presphenoid, zygomatic.right, ethmoid., maxilla.left, maxilla.right, lacrimal.left, lacrimal.right, palatine.left, palatine.right, inferior.nasal.concha.left, inferior.nasal.concha.right, vomer |
| 4 | 0.02967 | malleus.right, incus.right, stapes.right |
| 5 | 0.02967 | malleus.left, incus.left, stapes.left |
| 6 | 0.30854 | clavicle.right, scapula.right |
| 7 | 0.30854 | clavicle.left, scapula.left |
| 8 | 0.16817 | frontal.left, frontal.right, nasal.left, nasal.right, premaxilla.left, premaxilla.right |
| 9 | 1 | mandible.left, mandible.right |
| 10 | 1 | hyoid.bone |
| 11 | 1 | laryngeal.alar.cartilage |
| 12 | 1 | sternum |
| 13 | 1 | vertebrae |

**Tab. SI1-28.** Connectivity modules identified for the head of *Tupaia*.

| ID | p-value | Elements |
| --- | --- | --- |
| 1 | 0.00044 | frontal.left, frontal.right, occipital, parietal.left, parietal.right, sphenoid, temporal.left, temporal.right, zygomatic.left, zygomatic.right, mandible.left, mandible.right, vertebrae |
| 2 | 0.07865 | sternum, clavicle.left, scapula.left |
| 3 | 0.07865 | malleus.left, incus.left, stapes.left |
| 4 | 0.07865 | malleus.right, incus.right, stapes.right |
| 5 | 0.00885 | thyroid.cartilage, arytenoid.left, arytenoid.right, cricoid.cartilage |
| 6 | 0.30854 | clavicle.right, scapula.right |
| 7 | 3e-05 | ethmoid, lacrimal.left, lacrimal.right, maxilla.left, maxilla.right, nasal.left, nasal.right, palatine.left, palatine.right, premaxilla.left, premaxilla.right, vomer |
| 8 | 1 | hyoid.bone |

**Tab. SI1-29.** Connectivity modules identified for the head of *Cynocephalus*.

| ID | p-value | Elements |
| --- | --- | --- |
| 1 | 0.04332 | occipital, parietal.left, parietal.right, temporal.left, temporal.right, mandible.left, mandible.right, vertebrae |
| 2 | 0.07865 | sternum, clavicle.right, scapula.right |
| 3 | 0.07865 | malleus.right, incus.right, stapes.right |
| 4 | 0.07865 | malleus.left, incus.left, stapes.left |
| 5 | 0 | ethmoid, frontal.left, frontal.right, lacrimal.left, lacrimal.right, maxilla.left, maxilla.right, nasal.left, nasal.right, palatine.left, palatine.right, premaxilla.left, premaxilla.right, sphenoid, vomer, zygomatic.left, zygomatic.right |
| 6 | 0.00885 | thyroid.cartilage, arytenoid.left, arytenoid.right, cricoid.cartilage |
| 7 | 0.30854 | clavicle.left, scapula.left |
| 8 | 1 | hyoid.bone |

**Tab. SI1-30.** Connectivity modules identified for the head of *Lemur*.

| ID | p-value | Elements |
| --- | --- | --- |
| 1 | 0.01455 | occipital, parietal.left, parietal.right, temporal.left, temporal.right, mandible.left, mandible.right, vertebrae |
| 2 | 0.07865 | sternum, clavicle.right, scapula.right |
| 3 | 0.07865 | malleus.right, incus.right, stapes.right |
| 4 | 0.07865 | malleus.left, incus.left, stapes.left |
| 5 | 0 | ethmoid, frontal.left, frontal.right, lacrimal.left, lacrimal.right, maxilla.left, maxilla.right, nasal.left, nasal.right, palatine.left, palatine.right, premaxilla.left, premaxilla.right, sphenoid, vomer, zygomatic.left, zygomatic.right |
| 6 | 0.00885 | thyroid.cartilage., arytenoid.left, arytenoid.right, cricoid.cartilage |
| 7 | 0.30854 | clavicle.left, scapula.left |
| 8 | 1 | hyoid.bone |

**Tab. SI1-31.** Connectivity modules identified for the head of *Propithecus*.

| ID | p-value | Elements |
| --- | --- | --- |
| 1 | 0.07865 | sternum, clavicle.left, scapula.left |
| 2 | 0.07865 | malleus.left, incus.left, stapes.left |
| 3 | 0.07865 | malleus.right, incus.right, stapes.right |
| 4 | 0.02534 | occipital, parietal.left, parietal.right, temporal.left, temporal.right, mandible, vertebrae |
| 5 | 0 | ethmoid, frontal.left, frontal.right, lacrimal.left, lacrimal.right, maxilla.left, maxilla.right, nasal.left, nasal.right, palatine.left, palatine.right, premaxilla.left, premaxilla.right, sphenoid, vomer, zygomatic.left, zygomatic.right |
| 6 | 0.00885 | thyroid.cartilage, arytenoid.left, arytenoid.right, cricoid.cartilage |
| 7 | 0.30854 | clavicle.right, scapula.right |
| 8 | 1 | hyoid.bone |

**Tab. SI1-32.** Connectivity modules identified for the head of *Loris*.

| ID | p-value | Elements |
| --- | --- | --- |
| 1 | 0.01455 | occipital, parietal.left, parietal.right, temporal.left, temporal.right, mandible.left, mandible.right, vertebrae |
| 2 | 0.07865 | sternum, clavicle.right, scapula.right |
| 3 | 0.07865 | malleus.right, incus.right, stapes.right |
| 4 | 0.07865 | malleus.left, incus.left, stapes.left |
| 5 | 0 | ethmoid, frontal.left, frontal.right, lacrimal.left, lacrimal.right, maxilla.left, maxilla.right, nasal.left, nasal.right, palatine.left, palatine.right, premaxilla.left, premaxilla.right, sphenoid, vomer, zygomatic.left, zygomatic.right |
| 6 | 0.00885 | thyroid.cartilage., arytenoid.left, arytenoid.right, cricoid.cartilage |
| 7 | 0.30854 | clavicle.left, scapula.left |
| 8 | 1 | hyoid.bone |

**Tab. SI1-33.** Connectivity modules identified for the head of *Nycticebus*.

| ID | p-value | Elements |
| --- | --- | --- |
| 1 | 0.01455 | occipital, parietal.left, parietal.right, temporal.left, temporal.right, mandible.left, mandible.right, vertebrae |
| 2 | 0.07865 | sternum, clavicle.right, scapula.right |
| 3 | 0.07865 | malleus.right, incus.right, stapes.right |
| 4 | 0.07865 | malleus.left, incus.left, stapes.left |
| 5 | 0 | ethmoid, frontal.left, frontal.right, lacrimal.left, lacrimal.right, maxilla.left, maxilla.right, nasal.left, nasal.right, palatine.left, palatine.right, premaxilla.left, premaxilla.right, sphenoid, vomer, zygomatic.left, zygomatic.right |
| 6 | 0.00885 | thyroid.cartilage., arytenoid.left, arytenoid.right, cricoid.cartilage |
| 7 | 0.30854 | clavicle.left, scapula.left |
| 8 | 1 | hyoid.bone |

**Tab. SI1-34.** Connectivity modules identified for the head of *Tarsius*.

| ID | p-value | Elements |
| --- | --- | --- |
| 1 | 0.13415 | occipital, parietal.left, parietal.right, temporal.left, temporal.right, mandible, vertebrae |
| 2 | 0.07865 | malleus.right, incus.right, stapes.right |
| 3 | 0.07865 | malleus.left, incus.left, stapes.left |
| 4 | 0.00885 | thyroid.cartilage, arytenoid.left, arytenoid.right, cricoid.cartilage |
| 5 | 0.00028 | ethmoid, frontal, lacrimal.left, lacrimal.right, maxilla.left, maxilla.right, palatine.left, palatine.right, sphenoid, vomer, zygomatic.left, zygomatic.right |
| 6 | 0.30854 | clavicle.left, scapula.left |
| 7 | 0.30854 | clavicle.right, scapula.right |
| 8 | 0.09068 | nasal.left, nasal.right, premaxilla.left, premaxilla.right |
| 9 | 1 | hyoid.bone |
| 10 | 1 | sternum |

**Tab. SI1-35.** Connectivity modules identified for the head of *Callithrix*.

| ID | p-value | Elements |
| --- | --- | --- |
| 1 | 0.07865 | sternum, clavicle.left, scapula.left |
| 2 | 0.00151 | occipital, parietal.left, parietal.right, sphenoid, temporal.left, temporal.right, zygomatic.left, zygomatic.right, mandible, vertebrae |
| 3 | 0.07865 | malleus.right, incus.right, stapes.right |
| 4 | 0.07865 | malleus.left, incus.left, stapes.left |
| 5 | 4e-05 | ethmoid, frontal, lacrimal.left, lacrimal.right, maxilla.left, maxilla.right, nasal.left, nasal.right, palatine.left, palatine.right, premaxilla.left, premaxilla.right, vomer |
| 6 | 0.00885 | thyroid.cartilage, arytenoid.left, arytenoid.right, cricoid.cartilage |
| 7 | 0.30854 | clavicle.right, scapula.right |
| 8 | 1 | hyoid.bone |

**Tab. SI1-36.** Connectivity modules identified for the head of *Saimiri*.

| ID | p-value | Elements |
| --- | --- | --- |
| 1 | 0.07865 | sternum, clavicle.right, scapula.right |
| 2 | 0.00286 | occipital, parietal.left, parietal.right, sphenoid, temporal.left, temporal.right, zygomatic.left, zygomatic.right, mandible, vertebrae |
| 3 | 0.07865 | malleus.right, incus.right, stapes.right |
| 4 | 0.07865 | malleus.left, incus.left, stapes.left |
| 5 | 4e-05 | ethmoid, frontal, lacrimal.left, lacrimal.right, maxilla.left, maxilla.right, nasal.left, nasal.right, palatine.left, palatine.right, premaxilla.left, premaxilla.right, vomer |
| 6 | 0.00885 | thyroid.cartilage, arytenoid.left, arytenoid.right, cricoid.cartilage |
| 7 | 0.30854 | clavicle.left, scapula.left |
| 8 | 1 | hyoid.bone |

**Tab. SI1-37.** Connectivity modules identified for the head of *Aotus*.

| ID | p-value | Elements |
| --- | --- | --- |
| 1 | 0.07865 | sternum, clavicle.right, scapula.right |
| 2 | 0.00151 | occipital, parietal.left, parietal.right, sphenoid, temporal.left, temporal.right, zygomatic.left, zygomatic.right, mandible, vertebrae |
| 3 | 3e-05 | ethmoid, frontal, lacrimal.left, lacrimal.right, maxilla.left, maxilla.right, nasal.left, nasal.right, palatine.left, palatine.right, premaxilla.left, premaxilla.right, vomer |
| 4 | 0.07865 | malleus.left, incus.left, stapes.left |
| 5 | 0.07865 | malleus.right, incus.right, stapes.right |
| 6 | 0.00885 | thyroid.cartilage, arytenoid.left, arytenoid.right, cricoid.cartilage |
| 7 | 0.30854 | clavicle.left, scapula.left |
| 8 | 1 | hyoid.bone |

**Tab. SI1-38.** Connectivity modules identified for the head of *Pithecia*.

| ID | p-value | Elements |
| --- | --- | --- |
| 1 | 0.07865 | sternum, clavicle.left, scapula.left |
| 2 | 0.00094 | occipital, parietal.left, parietal.right, sphenoid, temporal.left, temporal.right, zygomatic.left, zygomatic.right, mandible, vertebrae |
| 3 | 0.07865 | malleus.right, incus.right, stapes.right |
| 4 | 0.07865 | malleus.left, incus.left, stapes.left |
| 5 | 3e-05 | ethmoid, frontal, lacrimal.left, lacrimal.right, maxilla.left, maxilla.right, nasal.left, nasal.right, palatine.left, palatine.right, premaxilla.left, premaxilla.right, vomer |
| 6 | 0.00885 | thyroid.cartilage, arytenoid.left, arytenoid.right, cricoid.cartilage |
| 7 | 0.30854 | clavicle.right, scapula.right |
| 8 | 1 | hyoid.bone |

**Tab. SI1-39.** Connectivity modules identified for the head of *Colobus*.

| ID | p-value | Elements |
| --- | --- | --- |
| 1 | 0.07865 | sternum, clavicle.right, scapula.right |
| 2 | 0.04702 | occipital, parietal.left, parietal.right, temporal.left, temporal.right, mandible, vertebrae |
| 3 | 0.07865 | malleus.right, incus.right, stapes.right |
| 4 | 0.07865 | malleus.left, incus.left, stapes.left |
| 5 | 0 | ethmoid, frontal, lacrimal.left, lacrimal.right, maxilla.left, maxilla.right, nasal.left, nasal.right, palatine.left, palatine.right, premaxilla.left, premaxilla.right, sphenoid, vomer, zygomatic.left, zygomatic.right |
| 6 | 0.00885 | thyroid.cartilage, arytenoid.left, arytenoid.right, cricoid.cartilage |
| 7 | 0.30854 | clavicle.left, scapula.left |
| 8 | 1 | hyoid.bone |

**Tab. SI1-40.** Connectivity modules identified for the head of *Cercopithecus*.

| ID | p-value | Elements |
| --- | --- | --- |
| 1 | 0.07865 | sternum, clavicle.right, scapula.right |
| 2 | 0.04702 | occipital, parietal.left, parietal.right, temporal.left, temporal.right, mandible, vertebrae |
| 3 | 0.07865 | malleus.right, incus.right, stapes.right |
| 4 | 0.07865 | malleus.left, incus.left, stapes.left |
| 5 | 0 | ethmoid, frontal, lacrimal.left, lacrimal.right, maxilla.left, maxilla.right, nasal.left, nasal.right, palatine.left, palatine.right, premaxilla.left, premaxilla.right, sphenoid, vomer, zygomatic.left, zygomatic.right |
| 6 | 0.00885 | thyroid.cartilage, arytenoid.left, arytenoid.right, cricoid.cartilage |
| 7 | 0.30854 | clavicle.left, scapula.left |
| 8 | 1 | hyoid.bone |

**Tab. SI1-41.** Connectivity modules identified for the head of *Papio*.

| ID | p-value | Elements |
| --- | --- | --- |
| 1 | 0.07865 | sternum, clavicle.right, scapula.right |
| 2 | 0.04702 | occipital, parietal.left, parietal.right, temporal.left, temporal.right, mandible, vertebrae |
| 3 | 0.07865 | malleus.right, incus.right, stapes.right |
| 4 | 0.07865 | malleus.left, incus.left, stapes.left |
| 5 | 0 | ethmoid, frontal, lacrimal.left, lacrimal.right, maxilla.left, maxilla.right, nasal.left, nasal.right, palatine.left, palatine.right, premaxilla.left, premaxilla.right, sphenoid, vomer, zygomatic.left, zygomatic.right |
| 6 | 0.00885 | thyroid.cartilage, arytenoid.left, arytenoid.right, cricoid.cartilage |
| 7 | 0.30854 | clavicle.left, scapula.left |
| 8 | 1 | hyoid.bone |

**Tab. SI1-42.** Connectivity modules identified for the head of *Macaca*.

| ID | p-value | Elements |
| --- | --- | --- |
| 1 | 0.07865 | sternum, clavicle.right, scapula.right |
| 2 | 0.04702 | occipital, parietal.left, parietal.right, temporal.left, temporal.right, mandible, vertebrae |
| 3 | 0.07865 | malleus.right, incus.right, stapes.right |
| 4 | 0.07865 | malleus.left, incus.left, stapes.left |
| 5 | 0 | ethmoid, frontal, lacrimal.left, lacrimal.right, maxilla.left, maxilla.right, nasal.left, nasal.right, palatine.left, palatine.right, premaxilla.left, premaxilla.right, sphenoid, vomer, zygomatic.left, zygomatic.right |
| 6 | 0.00885 | thyroid.cartilage, arytenoid.left, arytenoid.right, cricoid.cartilage |
| 7 | 0.30854 | clavicle.left, scapula.left |
| 8 | 1 | hyoid.bone |

**Tab. SI1-43.** Connectivity modules identified for the head of *Hylobates*.

| ID | p-value | Elements |
| --- | --- | --- |
| 1 | 0.07865 | sternum, clavicle.left, scapula.left |
| 2 | 0.04702 | occipital, parietal.left, parietal.right, temporal.left, temporal.right, mandible, vertebrae |
| 3 | 0.07865 | malleus.left, incus.left, stapes.left |
| 4 | 0.07865 | malleus.right, incus.right, stapes.right |
| 5 | 0 | ethmoid, frontal, lacrimal.left, lacrimal.right, maxilla.left, maxilla.right, nasal.left, nasal.right, palatine.left, palatine.right, premaxilla.left, premaxilla.right, sphenoid, vomer, zygomatic.left, zygomatic.right |
| 6 | 0.00885 | thyroid.cartilage, arytenoid.left, arytenoid.right, cricoid.cartilage |
| 7 | 0.30854 | clavicle.right, scapula.right |
| 8 | 1 | hyoid.bone |

**Tab. SI1-44.** Connectivity modules identified for the head of *Pongo*.

| ID | p-value | Elements |
| --- | --- | --- |
| 1 | 0.01868 | occipital, parietal.left, parietal.right, sphenoid, temporal.left, temporal.right, mandible, vertebrae |
| 2 | 0.07865 | malleus.right, incus.right, stapes.right |
| 3 | 0.07865 | malleus.left, incus.left, stapes.left |
| 4 | 0.00885 | thyroid.cartilage, arytenoid.left, arytenoid.right, cricoid.cartilage |
| 5 | 0.00064 | ethmoid, frontal, lacrimal.left, lacrimal.right, maxilla.left, maxilla.right, nasal.left, nasal.right, zygomatic.left, zygomatic.right |
| 6 | 0.30854 | clavicle.left, scapula.left |
| 7 | 0.30854 | clavicle.right, scapula.right |
| 8 | 0.25249 | palatine.left, palatine.right, vomer |
| 9 | 1 | premaxilla.left, premaxilla.right |
| 10 | 1 | hyoid.bone |
| 11 | 1 | sternum |

**Tab. SI1-45.** Connectivity modules identified for the head of *Gorilla*.

| ID | p-value | Elements |
| --- | --- | --- |
| 1 | 0.07865 | sternum, clavicle.right, scapula.right |
| 2 | 0.08452 | occipital, parietal.left, parietal.right, temporal.left, temporal.right, mandible, vertebrae |
| 3 | 0.07865 | malleus.right, incus.right, stapes.right |
| 4 | 0.07865 | malleus.left, incus.left, stapes.left |
| 5 | 0 | ethmoid, frontal, lacrimal.left, lacrimal.right, maxilla.left, maxilla.right, nasal.left, nasal.right, palatine.left, palatine.right, premaxilla.left, premaxilla.right, sphenoid, vomer, zygomatic.left, zygomatic.right |
| 6 | 0.00885 | thyroid.cartilage, arytenoid.left, arytenoid.right, cricoid.cartilage |
| 7 | 0.30854 | clavicle.left, scapula.left |
| 8 | 1 | hyoid.bone |

**Tab. SI1-46.** Connectivity modules identified for the head of *Pan troglodites*.

| ID | p-value | Elements |
| --- | --- | --- |
| 1 | 0.07865 | sternum, clavicle.right, scapula.right |
| 2 | 0.04702 | occipital, parietal.left, parietal.right, temporal.left, temporal.right, mandible, vertebrae |
| 3 | 0.07865 | malleus.left, incus.left, stapes.left |
| 4 | 0.07865 | malleus.right, incus.right, stapes.right |
| 5 | 2e-05 | sphenoid, zygomatic.left, zygomatic.right, frontal, ethmoidal, nasal.left, nasal.right, maxilla.left, maxilla.right, lacrimal.left, lacrimal.right, palatine.left, palatine.right, vomer |
| 6 | 0.00885 | thyroid.cartilage, arytenoid.left, arytenoid.right, cricoid.cartilage |
| 7 | 0.30854 | clavicle.left, scapula.left |
| 8 | 1 | premaxilla.left, premaxilla.right |
| 9 | 1 | hyoid.bone |

**Tab. SI1-47.** Connectivity modules identified for the head of *Pan paniscus*.

| ID | p-value | Elements |
| --- | --- | --- |
| 1 | 0.07865 | sternum, clavicle.right, scapula.right |
| 2 | 0.04702 | occipital, parietal.left, parietal.right, temporal.left, temporal.right, mandible, vertebrae |
| 3 | 0.07865 | malleus.left, incus.left, stapes.left |
| 4 | 0.07865 | malleus.right, incus.right, stapes.right |
| 5 | 2e-05 | sphenoid, zygomatic.left, zygomatic.right, frontal, ethmoidal, nasal.left, nasal.right, maxilla.left, maxilla.right, lacrimal.left, lacrimal.right, palatine.left, palatine.right, vomer |
| 6 | 0.00885 | thyroid.cartilage, arytenoid.left, arytenoid.right, cricoid.cartilage |
| 7 | 0.30854 | clavicle.left, scapula.left |
| 8 | 1 | premaxilla.left, premaxilla.right |
| 9 | 1 | hyoid.bone |

**Tab. SI1-48.** Connectivity modules identified for the head of Homo.

| ID | module | p-value | Elements |
| --- | --- | --- | --- |
| 1 | Left postcranial | 0.07865 | sternum, clavicle.left, scapula.left |
| 2 | Neurocranium, mandible, vertebrae | 0.04702 | occipital, parietal.left, parietal.right, temporal.left, temporal.right, mandible, vertebrae |
| 3 | Left ossicles | 0.07865 | malleus.left, incus.left, stapes.left |
| 4 | Right ossicles | 0.07865 | malleus.right, incus.right, stapes.right |
| 5 | Larynx vocal folds | 0.00885 | thyroid.cartilage, arytenoid.left, arytenoid.right, cricoid.cartilage |
| 6 | Viscerocranium | 1.00E-05 | sphenoid, zygomatic.left, zygomatic.right, frontal, ethmoidal, nasal.left, nasal.right, maxilla.left, maxilla.right, lacrimal.left, lacrimal.right, palatine.left, palatine.right, vomer |
| 7 | Right poscranium | 0.30854 | clavicle.right, scapula.right |
| 8 | hyoid | 1 | hyoid.bone |
